# Tailoring the stability/aggregation of one-dimensional TiO_2_(B)/titanate nanowires using surfactants

**DOI:** 10.3762/bjnano.10.103

**Published:** 2019-05-13

**Authors:** Atiđa Selmani, Johannes Lützenkirchen, Kristina Kučanda, Dario Dabić, Engelbert Redel, Ida Delač Marion, Damir Kralj, Darija Domazet Jurašin, Maja Dutour Sikirić

**Affiliations:** 1 Laboratory for Biocolloids and Surface Chemistry, Division of Physical Chemistry, Ruđer Bošković Institute, Bijenička Cesta 54, 10000 Zagreb, Croatia; 2 Karlsruhe Institute of Technology (KIT), Institute for Nuclear Waste Disposal, Hermann-von-Helmholtz-Platz 1, 76344 Eggenstein-Leopoldshafen, Germany; 3 Department of Chemistry, Faculty of Science, University of Zagreb, Horvatovac 102a, 10002 Zagreb, Croatia; 4 current affiliation: Department of Organic Chemistry, Weizmann Institute of Science, Rehovot 76100, Israel; 5 current affiliation: Department of Analytical Chemistry, Faculty of Chemical Engineering and Technology, Marulićev trg 19, 10000 Zagreb, Croatia; 6 Karlsruhe Institute of Technology (KIT), Institute of Functional Interfaces (IFG), Hermann-von-Helmholtz-Platz 1, 76344 Eggenstein-Leopoldshafen, Germany; 7 Center of Excellence for Advanced Materials and Sensing Devices, Institute of Physics, Bijenička 46, 10000 Zagreb, Croatia,; 8 Laboratory for Precipitation Processes, Division of Materials Chemistry, Ruđer Bošković Institute, Bijenička Cesta 54, 10000 Zagreb, Croatia

**Keywords:** 1D nanomaterials, cationic surfactants, stability, surface complexation model, titanate nanowires

## Abstract

The increased utilization of one-dimensional (1D) TiO_2_ and titanate nanowires (TNWs) in various applications was the motivation behind studying their stability in this work, given that stability greatly influences both the success of the application and the environmental impact. Due to their high abundance in aqueous environments and their rich technological applicability, surfactants are among the most interesting compounds used for tailoring the stability.

The aim of this paper is to determine the influence of surfactant molecular structure on TNW stability/aggregation behavior in water and aqueous NaBr solution by dynamic and electrophoretic light scattering. To accomplish this, two structurally different quaternary ammonium surfactants (monomeric DTAB and the corresponding dimeric 12-2-12) at monomer and micellar concentrations were used to investigate TNW stability in water and NaBr. It was shown that TNWs are relatively stable in Milli-Q water. However, the addition of NaBr induces aggregation, especially as the TNW mass concentration increases. DTAB and 12-2-12 adsorb on TNW surfaces as a result of the superposition of favorable electrostatic and hydrophobic interactions. As expected, the interaction of TNWs with 12-2-12 was stronger than with DTAB, due to the presence of two positively charged head groups and two hydrophobic tails. As a consequence of the higher adsorption of 12-2-12, TNWs remained stable in both media, while DTAB showed an opposite behavior.

In order to gain more insight into changes in the surface properties after surfactant adsorption on the TNW surface, a surface complexation model was employed. With this first attempt to quantify the contribution of the surfactant structure on the adsorption equilibrium according to the observed differences in the intrinsic log *K* values, it was shown that 12-2-12 interacts more strongly with TNWs than DTAB. The modelling results enable a better understanding of the interaction between TNWs and surfactants as well as the prediction of the conditions that can promote stabilization or aggregation.

## Introduction

Among the extensive variety of metal oxide nanomaterials, titanium dioxide nanomaterials (TNMs) (e.g., anatase, rutile, TiO_2_(B) and titanate) have attracted considerable attention because of their unique physicochemical properties compared to the bulk material. TNMs play an important role in various applications such as photocatalytic degradation of organic pollutants [1–2], sensors [3–4], solid oxide fuel cells [5], water purification [6–7], adsorption of radioactive and heavy metal ions [8], as well as antibacterial applications [9]. Their various applications can be divided into “energy” and “environment” related categories. Many of these applications as well as TNM interactions in the environment depend on their properties and modifications [10]. Therefore, increased application of TNMs has spurred numerous discussions and investigations concerning their behavior, transport and fate in aqueous environments [11–12].

The aggregation behavior, that is, the stability of nanomaterial (NM) dispersions in general, is one of the key factors for their successful application. Considering that the size of nano- and microaggregates greatly influences their degree of toxicity, and consequently their impact on human health and the environment, the conditions under which aggregation occurs are of interest for environmental but also for biomedical applications [13–14]. The stability of NM dispersions can be controlled by applying one of two approaches: (i) mechanical treatment or (ii) chemical and physical modification [15].

Recently, one-dimensional (1D) TNMs have emerged as an exceptional class of NMs. Their geometry offers unique properties that are difficult to achieve with other titanium oxide nanostructures [14,16]. It has been previously shown that various types of surface coatings affect NM properties, and in particular, can improve their stability and biocompatibility [13,17–19]. Although much is known about the aggregation of spherical TNMs in different media and in the presence of different additives [12,20–22], much less is known about 1D TNMs, such as titanium oxide nanotubes (TNTs), titanium oxide nanowires (TNWs) and titanium oxide nanorods (TNRs). As shown previously, the morphology of TNMs is expected to play a significant role in their stability, aggregation behavior and fate in aquatic environments [23]. As far as the stability of 1D TNWs is concerned, Szabó et al. [24] and Horváth et al. [25] investigated the surface charge and aggregation behavior of TNWs in the presence of polyelectrolytes (i.e., poly(styrene sulfonate) and poly(diallyldimethylammonium)chloride)). Their results show that oppositely charged polyelectrolytes strongly adsorb on TNW surfaces, leading to charge neutralization at the isoelectric point and subsequent charge reversal at higher polyelectrolyte concentration.

Apart from polyelectrolytes, another class of additives widely used for tailoring the stability of NMs are surfactants. The reason lies in their high tendency for adsorption, application in various industrial processes, synthesis of coated nanomaterials, etc. [26–29]. In addition, surfactants are widely used in households, and consequently, can be found in wastewater in high concentrations. Due to the high probability of the coexistence of surfactants and NMs in aqueous environments, studies on the effect of surfactants on the stability of NMs are essential. The presence of surfactants is expected to tremendously affect the stability of NMs, thereby altering their aggregation behavior as well as the ultimate bioavailability and eco-toxicity in aqueous environments [30–31].

Among the different classes of surfactants that have emerged in the last 30 years, dimeric, i.e., gemini surfactants have attracted particular attention in both fundamental research and industrial applications. Gemini surfactants possess a unique molecular structure involving two hydrophobic moieties connected by a hydrophobic or hydrophilic spacer at the level of the head groups. Consequently, these surfactants display superior physicochemical properties and distinctly different solution and interfacial behavior compared to corresponding conventional monomeric surfactants [32–34]. The interaction of gemini surfactants with solid (nano)surfaces such as clay [35], calcium phosphate [36], silica [37–40], TiO_2_ [41], ZnO [42] and carbon NTs [43] have been previously studied. With regard to TNWs, surfactants have been used in synthesis for the control of size and morphology [44–46], as well as for the synthesis of TNW membranes [47]. However, to the best of our knowledge, no study has been undertaken to determine the effect of surfactants on TNW stability in aqueous solutions.

In order to fill this void, in this study, the effect of monomers and micelles of (a) monomeric dodecyltrimethylammonium bromide (DTAB), and (b) its corresponding gemini, bis(*N,N*-dimethyl-*N*-dodecyl)ethylene-1,2-diammonium dibromide (12-2-12) quaternary ammonium surfactant (Scheme S1, Supporting Information File 1) on the stability of TNWs was compared. The motivation for such a choice of cationic surfactants was two-fold. On the one hand, TNWs are negatively charged above pH 4 [48], which is the pH range of interest in many applications, including environmental and biomedical. On the other hand, quaternary ammonium surfactants are the most commonly encountered cationic surfactants due to their high surface activity, good antibacterial properties as well as availability due to the ease of synthesis. In addition, comparing the effects of monomeric and corresponding gemini surfactants enables the effect of molecular and micellar structure to be determined while keeping surfactant chemistry the same [36]. It can be expected that surfactant adsorption on TNWs influences TNW stability by affecting the balance between the electrostatic, hydrophobic and steric interactions, similar to nanoparticle interactions with natural organic matter [49–51].

In this study the influence of TNW mass concentration, the effect of different surfactant molecular structures (number of positive head groups and hydrophobic chains) as well as the aggregation state (monomers and micelles) on the stability of TNWs was assessed in two media, water and aqueous electrolyte solution of sodium bromide, thus increasing the complexity of the investigated systems. The observed effects were quantified by surface complexation modeling (SCM) in order to describe the TNW behavior when surfactants adsorb onto the TNW surface. Moreover, the SCM in principle enables the prediction and optimization of surfactant adsorption on TNW surfaces, thus defining the conditions for stabilization or aggregation. The obtained results point to a simple way of controlling the TNW stability in dispersions and give insight into their possible behavior in the aqueous environment, opening a new opportunity for their safer application.

## Results

### Characterization of surfactants

The measured σ vs log *c* plots for DTAB and 12-2-12 (Figure S1, Supporting Information File 1) showed the typical reduction of surface tension with increase of surfactant concentration up to almost constant σ values, indicating the formation of micelles. As expected, 12-2-12 exhibited considerably lower σ values as well as a lower critical micelle concentration (cmc) than DTAB (Table S1 in Supporting Information) indicating its greater adsorption efficiency and stronger aggregating ability. The obtained results are in good agreement with literature data [32–33].

Based on the surface tension measurements, the surfactant concentrations reflecting different aggregation states (monomers and micelles) were chosen.

### Characterization of TNWs

The mixed phase, TiO_2_(B) and trititanate layered TNW structure was confirmed by powder X-ray diffraction (PXRD) as well as Fourier-transform infrared spectroscopy (FTIR) and Raman spectroscopy. The details can be found in Supporting Information File 1.

**Figure 1:**
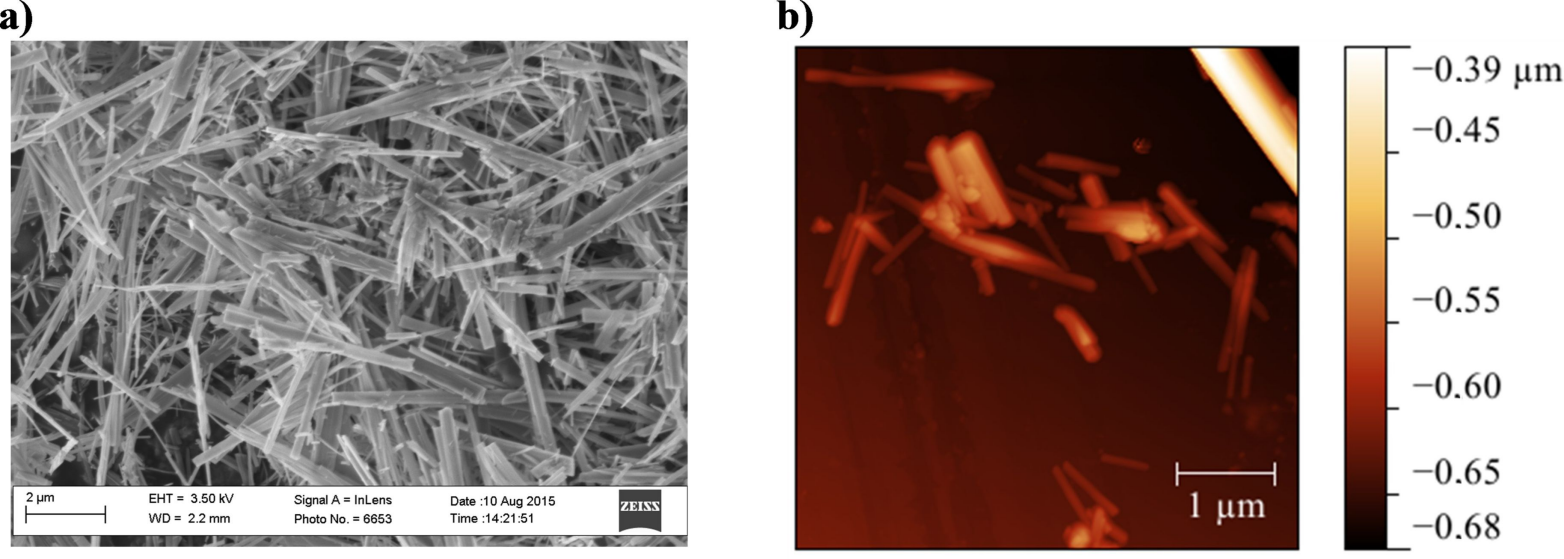
Micrographs of synthesized TNWs taken by a) high-resolution scanning electron microscopy and b) atomic force microscopy.

High-resolution scanning electron microscopy (HR-SEM) micrographs revealed that the synthesized TNWs have a distinct, straight, wire-like morphology (Figure 1a). The analysis of the micrographs showed that the length of the TNWs is in the range from 900 to 2000 nm, while the measured diameter ranged from 25 to 250 nm. Atomic force microscopy (AFM) revealed the tendency of the material to form bundles from a pair of (or more) TNWs (Figure 1b). However, single TNWs were also visible. From the AFM topographs the diameter of a single TNW has been estimated to vary between 25 and 175 nm, while the length varied from rather short fragments (50 nm) to much longer TNWs of 800 nm. The specific surface area of the TNWs, determined from nitrogen adsorption isotherms (BET), was equal to 24.1 m^2^ g^−1^.

**Figure 2:**
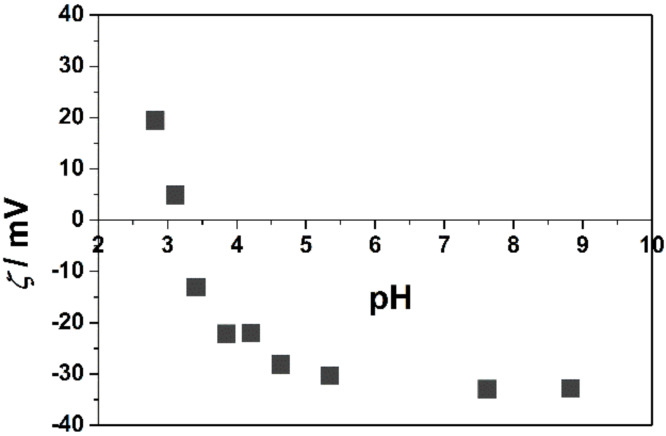
Variation of zeta potential (ζ) with pH of TNWs in 10^−3^ mol dm^−3^ NaBr aqueous solution. γ(TNW) = 1 × 10^−2^ g dm^−3^. θ = 25 °C.

The surface of the TNWs consists of active surface groups that can be either uncharged, positive or negatively charged depending on the pH of the solution. The isoelectric point, pH_iep_ of the TNWs determined from the zeta potential measurements was found to be pH 3.2, as shown in Figure 2. This means that in the investigated pH region (pH > 3.2) bare TNWs are negatively charged.

### Stability of TNWs and TNW/surfactant dispersions

The stability of the materials is of utmost importance for the successful application of NMs in general, as well as for their fate in the environment. Many applications require stable NM dispersions, while for some others, aggregation may be desirable. In aqueous medium, amphoteric surface groups of metal oxide NMs can be protonated or deprotonated depending on the pH values, which gives rise to a surface charge compensated by counter-ion. As a consequence an electrical double layer (EDL) is formed [52]. According to classical Derjaguin–Landau–Verwey–Overbeek (DLVO) theory, the stability of the NMs is determined by two major contributions: the repulsive double layer interaction potential (overlapping EDL) and the attractive van der Walls force [53–54]. The average hydrodynamic diameter can be reduced as the zeta potential increases, due to enhanced repulsive electrostatic force and particle stabilization.

#### Effect of TNW concentration on the stability of TNW dispersions

In this study the stability was followed in dispersions at three different TNW concentrations (γ/g dm^−3^ = 1 × 10^−2^ (CS1), 5 × 10^−2^ (CS2), 1 × 10^−1^ (CS3)) by monitoring changes in size (*d*_h_) and zeta potential (ζ) over 24 h as represented in Table 1 and Figure S8a–d, Supporting Information File 1. At the lowest TNW concentration (CS1), the TNW dispersions were stable during 24 hours, as indicated by an almost constant *d*_h_ at around 335 nm. With increasing TNW concentration, CS2 and CS3, *d*_h_ increased compared to CS1 to 670 nm and 550 nm, respectively. At low mass concentration, the TNWs remained stable due to the larger distance between particles, compared to the CS2 and CS3 systems [22]. A further increase of the TNW mass concentration of particles often leads to increased collision frequency, thus facilitating aggregation. Although the size of the aggregates changed as the TNW mass concentration increased, the zeta potential was almost the same for all CS samples, confirming the stability of dispersions. Unlike for the majority of small spherical NMs, the obtained results suggest that aqueous dispersions of TNWs may be rather stable even without stabilizing agents. It is well-established that the concentration of NMs may have a significant effect on the dispersion stability [22,55]. In contrast, Hsiung et al. [56] concluded that the stability of some commercial TiO_2_ NMs was independent of their concentration in the concentration range of 5 × 10^−2^ to 2 × 10^−1^ g dm^−3^. The addition of NaBr increased the *d*_h_, which was more pronounced in CS2 (840 nm) and CS3 (950 nm) as represented in Table 1 and Figure S8c, Supporting Information File 1. In the presence of NaBr, the zeta potential of the TNWs in all CS samples became slightly more negative as compared to the systems without NaBr. The observed results might be explained in terms of compression of the EDL, which in our results becomes more pronounced as the mass concentration of TNWs increases, thus enabling aggregation of TNWs.

**Table 1:** The *d*_h_ and and zeta potential (ζ) of the bare TNWs in Milli-Q water and 1 × 10^−3^ mol dm^−3^ NaBr after 24 h. γ/g dm^−3^ = 1 × 10^−2^ (CS1), 5 × 10^−2^ (CS2), 1 × 10^−1^ (CS3). θ = 25 °C.

Control system	Milli-Q water		NaBr	
	*d*_h_ / nm	ζ / mV	*d*_h_ / nm	ζ / mV

CS1	333.8 ± 36.1	−30.8 ± 2.8	455.2 ± 93.8	−32.5 ± 2.8
CS2	669.1 ± 78.1	−29.3 ± 1.3	832.8 ± 89.0	−35.2 ± 0.3
CS3	547.3 ± 84.8	−32.8 ± 0.7	943.6 ± 56.2	−38.8 ± 1.0

Similarly, Szabó et al. [24] showed that increasing the salt content leads to faster aggregation of the bare TNWs. The obtained results point to a delicate interplay between mass and salt concentration, which could change TNW stability.

#### Effect of surfactant concentration on the stability of TNW dispersions

Since TNWs are negatively charged above pH 4, in this study, monomer and micellar concentrations of the cationic monomeric DATB and corresponding gemini 12-2-12 surfactants (Figure S1, Supporting Information File 1) were chosen as a tool to modify the TNW surface properties.

**Figure 3:**
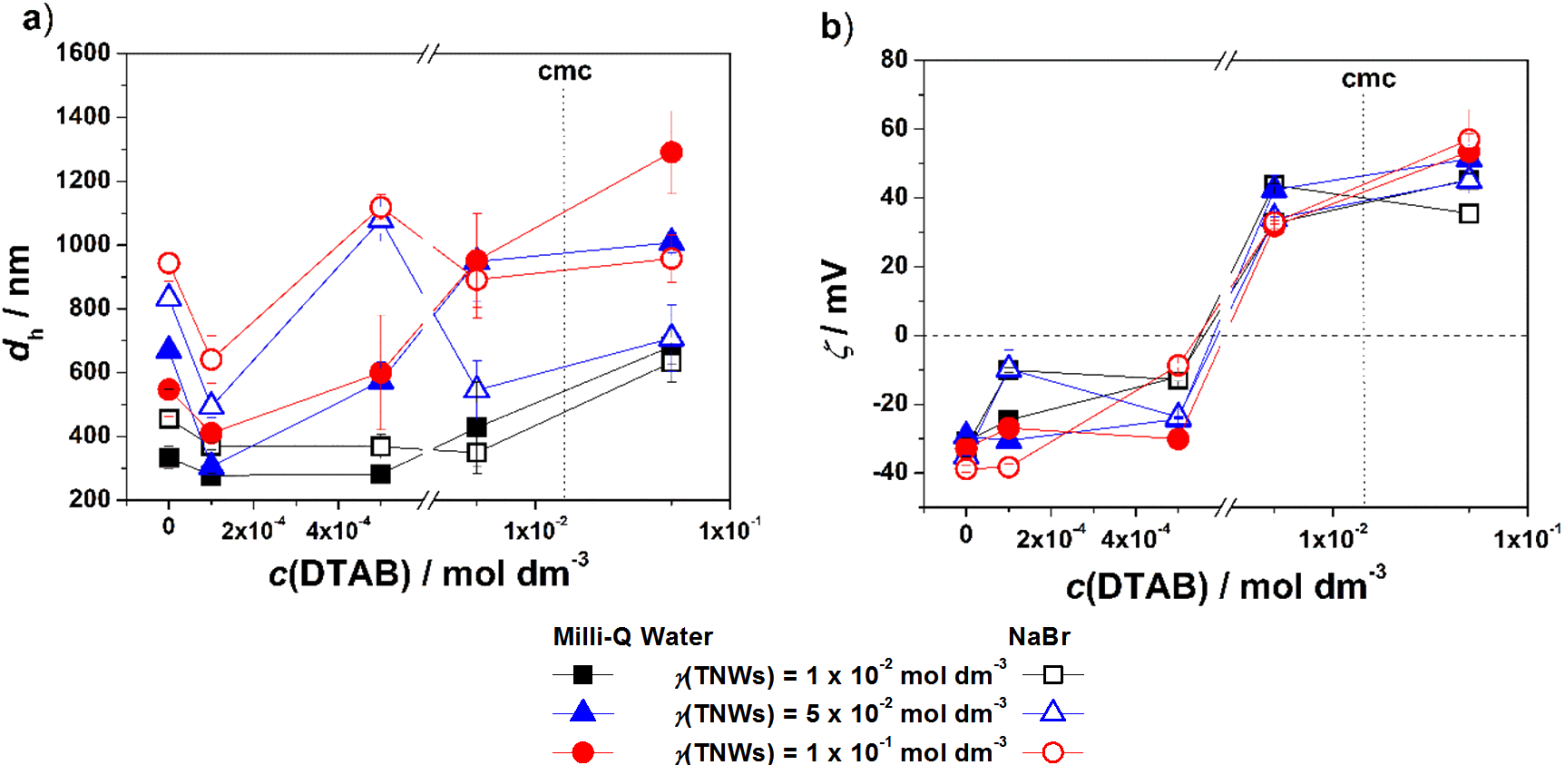
The *d*_h_ (a) and (b) zeta potential (ζ) of DTAB/TNWs in Milli-Q water and 1 × 10^−3^ mol dm^−3^ NaBr after 24 h. γ(TNW)/g dm^−3^ = 1 × 10^−2^, 5 × 10^−2^, 1 × 10^−1^. θ = 25 °C. The critical micelle concentration (cmc) of DTAB is marked by the dotted line. The lines connecting the data points are a guide for the eye.

**Effect of DTAB concentration:** The variation of *d*_h_ and the zeta potential in TNW/DTAB systems at three TNW mass concentrations after 24 h are shown in Figure 3a,b. The results of the visual monitoring are presented in Figure S6, Supporting Information File 1. The *d*_h_ and zeta potential monitored during 24 h are presented in Supporting Information File 1, Figures S9 and S10. In the presence of the lowest investigated DTAB concentration (A1, B1 and C1), differences in *d*_h_ and zeta potential of TNWs due to the increase of their concentration were smaller than that observed for the control systems (Figure 3). When NaBr (A1*, B1*, C1*) was added, the *d*_h_ of the TNWs increased, with the effect being more pronounced at the highest TNW concentration. The further increase of the DTAB concentration, *c*/mol dm^−3^ = 5 × 10^−4^ (A2, B2 and C2), leads to an increased *d*_h_ in B2 and C2. For the system A2, a lower absolute zeta potential is detected, while in B2 and C2, no change in zeta potential was observed. This is due to the fact that at higher mass concentrations more DTAB is needed to affect the surface charge and consequently the zeta potential.

The presence of NaBr promoted the aggregation of dispersions with higher TNW mass concentration. A decrease in the absolute value of the zeta potential was observed and less stable TNW dispersions were obtained. At DTAB concentrations 5 × 10^−3^ (the highest monomer concentration) and 5 × 10^−2^ mol dm^−3^ (micellar concentration) the *d*_h_ and zeta potential of the systems increased. The change of *d*_h_ was more pronounced with increasing TNW concentration. This is due to surface charge neutralization when positively charged head groups adsorb and neutralize negatively charged TNW surfaces, which is followed by the interaction between hydrophobic tails of adsorbed surfactant molecules and those in the bulk causing charge reversal (Figure 3a,b). The charge reversal indicates the formation of surfactant bilayer structures at the surface of TNWs. The zeta potential measured in the systems with the highest DTAB concentrations (A3, A4, B3, B4, C3 and C4) were higher than 30 mV, even though the size of the aggregates was close to 1 μm or larger. In these systems (A3*-A4*, B3*-B4*, C3*-C4*), the presence of NaBr affected neither the *d*_h_ nor the zeta potential.

**Figure 4:**
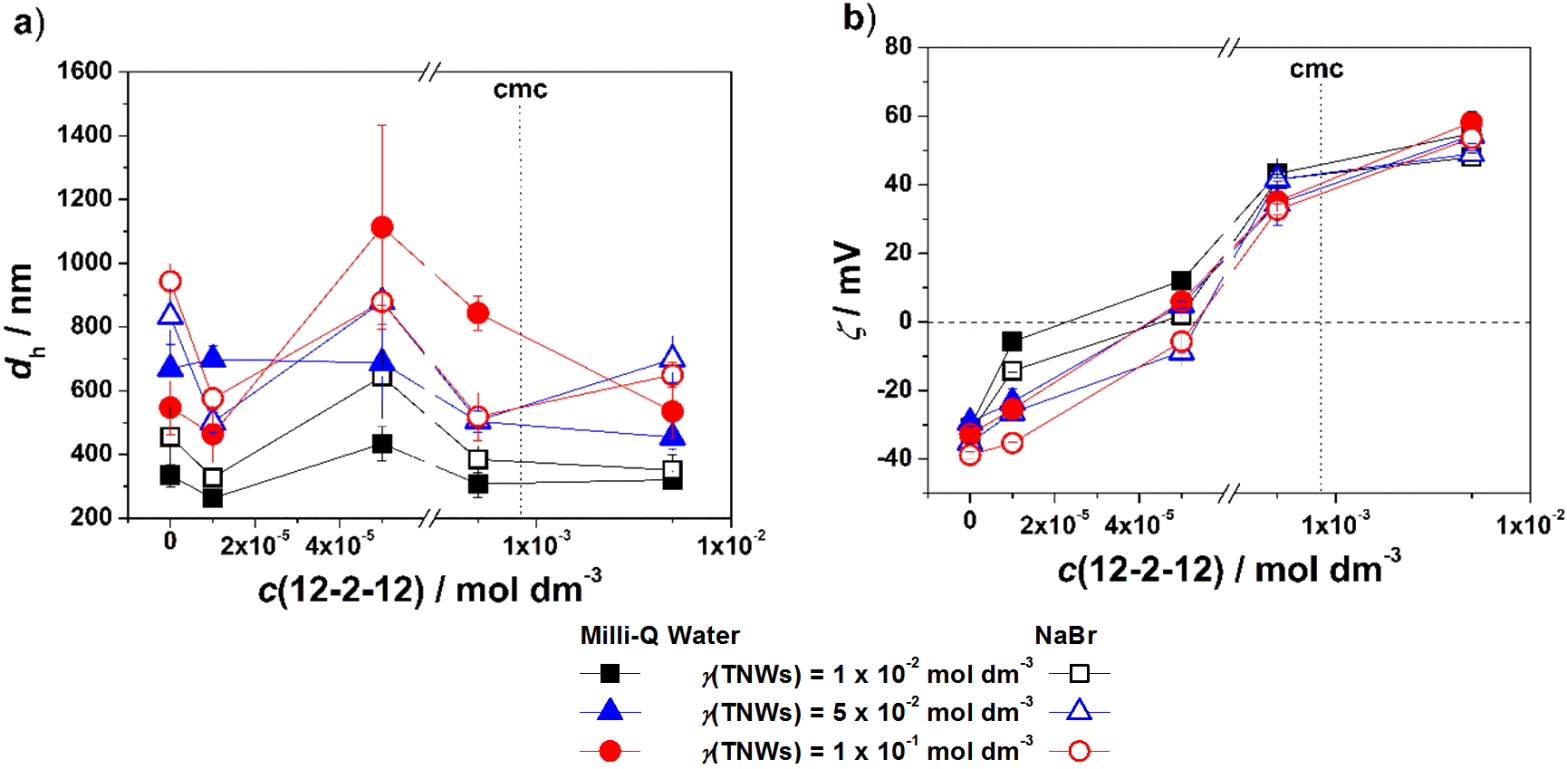
The *d*_h_ (a) and (b) the zeta potential (ζ) of 12-2-12/TNWs in Milli-Q water and 1 × 10^−3^ mol dm^−3^ NaBr after 24 h. γ(TNW)/g dm^−3^ = 1 × 10^−2^, 5 × 10^−2^, 1 × 10^−1^. θ = 25 °C. The critical micelle concentration (cmc) of 12-2-12 is indicated with dotted line. The lines connecting the data points are a guide for the eye.

**Effect of 12-2-12 concentration:** The results obtained by dynamic light scattering (DLS) and electrophoretic light scattering (ELS) after 24 h are represented in Figure 4a,b. In Supporting Information File 1, the visual inspection results (Figure S7) as well as *d*_h_ and the zeta potential results monitored during 24 h are presented (Figures S11a–f and S12a–f). The *d*_h_ values obtained at the lowest monomeric 12-2-12 concentration (D1, E1 and F1) were similar to those measured in the corresponding control systems (CS1-3). The measured zeta potentials were more positive, especially in D1, due to adsorption of 12-2-12 molecules, as in the case of DTAB. The presence of NaBr in these dispersions (D1*, E1* and F1*) resulted in the formation of larger aggregates. At higher monomeric 12-2-12 concentrations (D2, E2 and F2), more 12-2-12 molecules are adsorbed on the TNW surface and *d*_h_ increases.

For these systems, a decrease in the absolute value of the zeta potential was observed, resulting in suppressed electrostatic repulsion between particles. The attractive forces started to dominate and led to TNW aggregation. The observed effect was most significant in the F2 system. The addition of NaBr destabilized the dispersions with a lower TNW concentration (D2*, E2*), as evidenced by increased *d*_h_. However, the change in the zeta potential was not that pronounced. The increase of the 12-2-12 concentration resulted in more stable dispersions, which was confirmed by lower *d*_h_ values and increased zeta potential in the D3-D4, E3-E4 and F3-F4 systems as compared to D2, E2 and F2. In these systems (D3*-D4*, E3*-E4*, F3*-F4*), the presence of NaBr affected *d*_h_ (when compared to corresponding Milli-Q water systems) even though the zeta potential values were positive, indicating stable dispersions.

**Figure 5:**
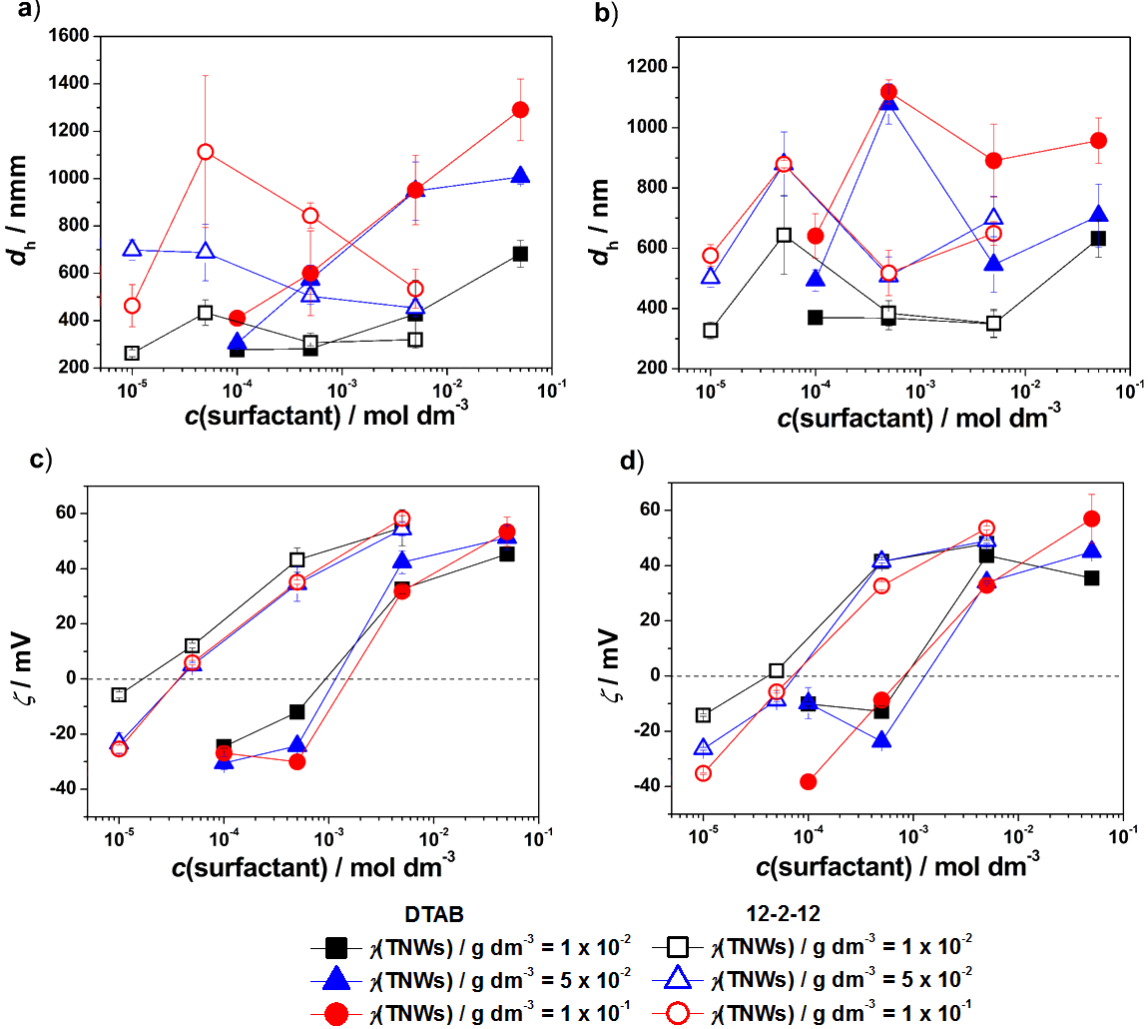
The comparison of *d*_h_ obtained for TNW/surfactant systems in Milli-Q water (a) and 1 × 10^−3^ mol dm^−3^ NaBr (b) and the zeta potential (ζ) in Milli-Q water (c) and 1 × 10^−3^ mol dm^−3^ NaBr (d) after 24 h. θ = 25 °C. The lines connecting the data points are a guide for the eye.

The comparison of the stabilization effect and adsorption ability for both surfactants onto TNW surfaces in Milli-Q water and NaBr aqueous electrolyte solution is shown in Figure 5a–d.

The increasing DTAB concentration in TNW suspensions in Milli-Q water resulted in increased *d*_h_ at all investigated TNW concentrations. In contrast, the increasing 12-2-12 concentration resulted in a decrease of *d*_h_ indicating a stabilizing effect. In addition, the obtained *d*_h_ values were smaller than those in TNW/DTAB systems. Unlike the constant increase of *d*_h_ with DTAB concentration, in the presence of 12-2-12, the largest *d*_h_ values were observed at *c*(12-2-12)/mol dm^−3^ = 5 × 10^−5^. In NaBr aqueous electrolyte solution, the aggregation of TNWs was promoted. In TNW/DTAB systems the largest increase of *d*_h_ was observed at *c*(DTAB)/mol dm^−3^ = 5 × 10^−4^, while for TNW/12-2-12 systems at *c*(12-2-12)/mol dm^−3^ = 5 × 10^−5^. The highest monomer concentration of DTAB (5 × 10^−3^ mol dm^−3^) and 12-2-12 (5 × 10^−3^ mol dm^−3^) leads to more stable TNWs. The positive value of the zeta potential in both cases increases as the concentration of surfactants increases. The only significant difference between DTAB and 12-2-12 is the concentration at which charge reversal is observed. For 12-2-12 charge reversal was detected at 100 times lower 12-2-12 concentration, that is *c*(12-2-12)/mol dm^−3^ = 5 × 10^−5^, while for DTAB charge reversal was detected at 5 × 10^−3^ mol dm^−3^. The obtained results lead to the conclusion that the stabilization effect of the surfactants on TNWs is strongly dependent on the composition of the media (pH, ionic strength), TNW mass concentration as well as the molecular structure of the surfactants used.

#### pH Titrations and surface complexation modelling

**Figure 6:**
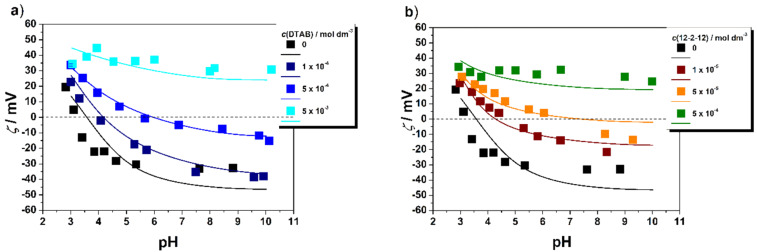
The zeta potential (ζ) of TNWs with and without surfactant in NaBr aqueous electrolyte solution, *c*(NaBr)/mol dm^−3^ = 1 × 10^−3^ and at different a) DTAB concentrations, *c*(DTAB)/mol dm^−3^ = (black square) 0, (dark blue square) 1 × 10^−4^, (blue square) 5 × 10^−4^, (light blue square) 5 × 10^−3^; b) *c*(12-2-12)/mol dm^−3^ = (black squre) 0, (red square) = 1 × 10^−5^, (orange square) 5 × 10^−5^, (green square) 5 × 10^−4^. γ(TNW)/g dm^−3^ = 1 × 10^−2^ , θ = 25 °C. The modelling results of TNW zeta potential measurements with and without surfactant in NaBr aqueous electrolyte solution, *c*(NaBr)/mol dm^−3^ = 1 × 10^−3^ are shown as the corresponding solid lines. The experimental results were fitted using surface complexation modeling (SCM).

The influence of pH on the TNW/DTAB and TNW/12-2-12 systems was investigated in order to determine the effect of the surfactants on the surface charging in the respective dispersions. The measured variations of the zeta potential with pH are shown in Figure 6a,b. The two lowest monomer concentrations of both surfactants shifted the pH_iep_ to higher pH and resulted in less negative values of the zeta potential, thus confirming the adsorption of the surfactants on the TNWs. The shift in pH_iep_ is more prominent for the 12-2-12 due to two positively charged head groups, which caused stronger electrostatic interactions with TNWs. The adsorption of the highest investigated monomer concentration of DTAB and 12-2-12 on TNWs led to charge reversal and the zeta potential became positive over the entire pH region studied.

In order to gain insight into the adsorption of the applied surfactants on the TNW surface and the respective interfacial equilibria, a surface complexation model (SCM) was designed based on the one previously used to describe TNW charging in the absence of surfactants [48].

Not many surface complexation models involving surfactant adsorption are available. A recent example in the work of Tagavifar et al. involves a purely diffuse double layer model [57]. Here, we use a more complex model. The surface complexation model starts from the previous model developed for the bare TNWs. These fundamental charging settings are given in the first three lines of Table 2. Based on this, the zeta potential measurements in the presence of the two surfactants were used to obtain the simplest possible option that would describe the experimental data. The basic Stern model is used to define the interface in terms of planes of adsorption. These are the 0- and β-planes, where in the 0-plane protons are adsorbed, and in the β-plane the electrolyte ions are bound as point charges, i.e., no charge distribution was considered. In the previous study a slip plane parameter was required to model the zeta potential [48]. In the present study, we were interested to see to what extent this parameter would change with the presence of the surfactant molecules and whether a constant slip plane parameter would allow the data to be described with different total surfactant concentrations.

**Table 2:** Surface complexation parameters for a tentative description of the electro-kinetic data. TTT is an acronym for 12-2-12. The slip plane parameter is the relation between the slip plane distance and the Debye length. A value of zero would mean that the slip plane coincides with the head-end of the diffuse layer. A value of 0.5 means that the slip plane is at half the Debye length for the respective ionic strength of the 1:1 electrolyte. Here, this concentration is 1 mM and the Debye length at 25 °C is 9.6 nm.

Reaction equation	log *K*	*C* / Fm^−2^	Slip plane parameter

≡ TiOH^−1/2^ + H^+^ ↔ ≡ TiOH_2_^+½^	3.74		
≡ TiOH^−1/2^ + C^+^ ↔ ≡ TiOH^−1/2^ · C^+^	−0.20	0.7	0.7
≡ TiOH^+1/2^ + A^−^ ↔ ≡ TiOH^+1/2^ · A^–^	−0.64		
≡ TiOH^−1/2^ + DTAB^+^ ↔ ≡ TiOH_2_^+1/2^ · DTAB^+^	3.00		0.4
≡ TiOH^−1/2^ + TTT^2+^ ↔ ≡ TiOH^−1/2^ · TTT^2+^	3.95		0.3

**Figure 7:**
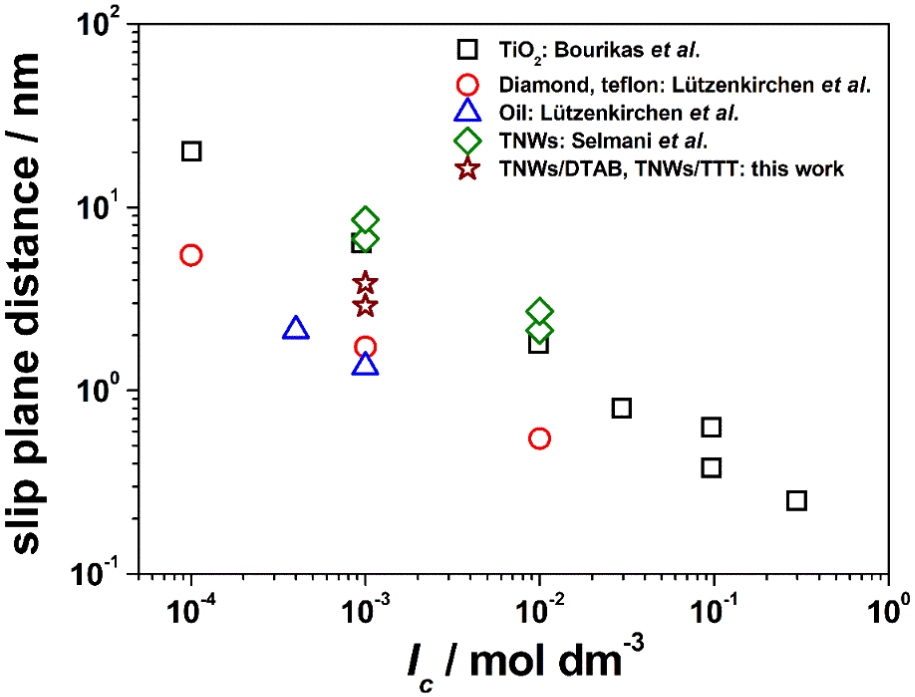
Best fit slip plane distances as a function of ionic strength. The data are taken from the literature. TiO_2_ from Bourikas et al. [58] (black squares), diamond, Teflon (red circles) and oil (blue triangles) are taken from Lützenkirchen et al. [59], TiO_2_ nanowires from Selmani et al. [48] (green diamonds), and data determined in the frame of the current investigation (brown stars) are shown.

The final model nicely fits the experimental zeta potential data. In particular, when the total concentration of the surfactants are changed, the model describes the charge inversion or the condition where no pH-dependent variation occurs. The reaction stoichiometry is the same for the two surfactants and both are treated as ideal outer-sphere complexes as far as the charged head-groups are concerned. Therefore, the intrinsic log *K* values can be used to compare their affinity for the nanowires. The binding of the 12-2-12 is clearly stronger. Interestingly, compared to the "ideal" slip plane parameter in the absence of surfactant, the slip plane distance is smaller by about 50% on average. If the slip plane in the absence of surfactant were to be mechanistically meaningful, this would suggest that the surfactant would perturb the interfacial layer in the sense that the structure is weakened by the hydrophobic tails. However, the slip plane parameter is probably best viewed as a fitting parameter. Figure 7 shows that the slip plane parameter determined for the bare TNW surfaces [48] agrees with parameters previously reported for different TiO_2_ polymorphs by Bourikas et al. [58]. The values for hydrophobic surfaces are from Lützenkirchen et al. [59].

The fitted values for the TNWs in the presence of surfactants are intermediate between the bare TNWs and two sets for the purely hydrophobic surfaces. While the parameters appear self-consistent in going from a hydrophilic to a hydrophobic system, it cannot be excluded that the outcome in terms of the slip plane distances is accidental. Thus, another hydrophobic system (the air/water interface) has fitted slip plane distances that coincide with those of the bare minerals. We note that the slip plane distances for the low ionic strength appear excessive. However, the amount of oriented water as observed in sum frequency generation studies also strongly decreases with increasing ionic strength in the absence of surfactants [60].

Within the SCM model framework, the observed surfactant behavior when adsorbed on TNWs was tentatively correlated with their molecular structure. The obtained intrinsic log *K* values give insights into the interaction between surfactants and the TNW surface. The 12-2-12 is assigned as the surfactant that has higher affinity for the TNW surface, which was confirmed with the experimental results.

## Discussion

Interactions between NMs and surfactants are becoming increasingly important not only in different technologies, but also in environmental protection, due to the widespread use of both types of materials. Adding to the importance of studying these interactions is the fact that their final outcome is not as easily predicted as in the case of simple ions or simple organic molecules. The reason lies in the fact that adsorption of surfactants is the result of both electrostatic and hydrophobic interactions [40], which are influenced by experimental conditions such as pH, ionic strength, concentration of NMs as well as the surfactants in different ways.

Surfactant adsorption at the solid/solution interface was used to modify the surface of TNWs and hence influence their colloidal stability. As a direct consequence of their amphiphilic nature, surfactant adsorption behavior differs significantly from that of small molecules and ions. During the adsorption of the surfactant molecules on oppositely charged solid surfaces, several steps can be recognized on an adsorption isotherm.

Several models for adsorption of surfactants on solid/aqueous interfaces have been proposed but the most widely accepted one for the cationic surfactants is the four-step or reverse orientation model [40]. Briefly, according to that model, the adsorption of surfactants is governed by electrostatic and hydrophobic interactions. Surfactants adsorb already at low concentrations due to electrostatic interactions between their charged head groups and the oppositely charged solid surface. As the surfactant concentration increases, so do the hydrophobic interactions between hydrophobic tails of the adjacent adsorbed surfactant molecules. In this step the combined effect of electrostatic and hydrophobic interactions is a sharp increase in the adsorption density. After the solid surface is fully covered by the surfactant, i.e., electrically neutral, further adsorption is governed by hydrophobic interactions and surface aggregates – hemi-micelles are formed resulting in the increase of the adsorption density. Ultimately, a surface bilayer in which the heads of the second surfactant layer are oriented towards the solution forms and the maximum absorption density is achieved. Any further increase in the surfactant concentration (above the cmc) leads only to formation of micelles in the solution [36,40].

From the results obtained in this study it can be concluded that the adsorption of the selected surfactants on TNW surfaces follows some general principles. When suspended in pure water uncoated TNWs are negatively charged due to the deprotonation of surface groups. The negative charge enables favorable interaction of the TNW surface groups with positively charged surfactant molecules. Hence, at lower surfactant concentrations, electrostatic interactions dominate. Consequently, adsorption of DTAB and 12-2-12 results in neutralization of the negative surface charge and less negative zeta potential values of the TNWs. As expected, as the surfactant concentration increased, the zeta potential of the TNWs approached zero and interparticle repulsions were reduced as well as the stability of the dispersions. The addition of NaBr affects the aggregation in these systems. The increase in ionic strength leads to a screening effect of the surfactant charge by bromide ions and the repulsive forces are reduced, thus promoting aggregation. In the systems with higher concentrations of DTAB and 12-2-12, charge reversal was observed by the zeta potential measurements. Positive zeta potential values indicate that head groups of surfactant molecules adsorbed on TNW are facing towards the solution, due to the formation of the bilayer. The formation of the bilayers at a surfactant concentration below the cmc reflects the high adsorption affinity of the chosen surfactants for the TNW surface and strong hydrophobic interactions. The addition of NaBr did not influence the size and zeta potential of TNW dispersions due to high coverage of the TNW surface with surfactant molecules and formation of bilayers. Further increase in the surfactant concentration, i.e., addition of micellar DTAB and 12-2-12 concentration resulted in more positive charge of TNWs as seen from the zeta potential measurements. Hydrophobic interactions between surfactant tails become a major driving force at this stage of surfactant adsorption. The highest concentration of both DTAB and 12-2-12 increases the TNW size. The zeta potential of the particles in these systems had the largest absolute values, which strongly indicates electrostatic TNW stabilization by micellar DTAB and 12-2-12 concentrations. The presence of NaBr in the TNW dispersions does not exhibit an effect on *d*_h_ and zeta potential values. The obtained results indicate how the stability of TNW dispersions can be changed by varying the surfactant concentration.

In the light of the literature data, we can conclude that the stability/aggregation of TNW dispersions cannot be simply quantified by an interplay between electrostatic repulsive and hydrophobic attractive interactions. Similar results for adsorption of conventional and respective gemini surfactants onto soil particles were reported by Rosen and Li [61], Fan et al.[62] and Dobson et al. [63]. The charge reversal in the zeta potential of TNWs occurred at concentrations two orders of magnitude lower in systems with 12-2-12 compared with DTAB. If we suppose that the second head group of 12-2-12 is not bound at the surface, as shown in work of Grosmarie et al. [64], the obtained results can be attributed to the higher adsorption affinity and stronger hydrophobic interaction in systems with dimeric surfactant due to the presence of two dodecyl chains*.* The presence of DTAB and 12-2-12 affects the surface charge of the TNWs. Both surfactants showed a shifted pH_iep_ of TNWs to higher pH values, indicating the adsorption of the DTAB and 12-2-12. With further increase of the surfactant concentration, charge reversal was obtained and zeta potential was positive regardless of the pH. The comparison of the respective DTAB and 12-2-12 effects on TNW stabilization reveals that the adsorption of DTAB and 12-2-12 onto the TNW surface was affected by media composition, TNW mass concentration, molecular structure and concentration of surfactants. 12-2-12 was a better choice for the manipulation of the TNW stability/aggregation under the given conditions. Therefore, it can be concluded that lower concentrations of 12-2-12 can be used either for stabilization or aggregation of TNWs compared with DTAB.

Similar results were obtained by Veronovski et al. where the stabilization of TiO_2_ P25 dispersions was tailored by 12-6-12 concentrations below the critical micelle concentration [27]. The TNW mass concentration exhibits an additional effect of promoting aggregation. The TNW dispersions were less stable as the TNW concentration increased, for all investigated systems. The effect of NaBr on TNW stability depends on the surfactant concentration, i.e., it depends on TNW surface coverage with surfactants. Due to the screening effect, at lower surfactant concentrations, when surface coverage is incomplete, aggregation is more pronounced.

The SCM approach was applied to simulate surfactant adsorption on the TNW surface in order to quantify the contribution of the surfactant structure on the adsorption equilibrium. In the literature, there are not many studies where a comprehensive experimental and theoretical approach was applied. The most recent one is by Tagavifar et al. [57] where the pH effect on anionic surfactant adsorption on limestone was studied in order to investigate the dynamics of surfactant adsorption. To the best of our knowledge, for the first time in our study the SCM model was used to describe the experimental data for oxidic systems. The modelling results confirm that 12-2-12 has a higher intrinsic log *K*, thus confirming that 12-2-12 interacts more strongly with TNW surfaces when compared with DTAB. The reason for this behavior might be the different structure of adsorbed surfactant layers, which is a consequence of their different molecular structure. The model enables prediction of zeta potential values as a function of pH, TNW concentration, salt level and surfactant concentration, providing a way to tailor the stability of TNW dispersions and enabling better understanding of the surfactant behavior in contact with TNW surfaces.

## Conclusion

The stability of the TiO_2_ nanoparticles, i.e., the size of nano- and microaggregates in aqueous environment, is known to be affected by their concentration, pH, electrolytes and the presence of organic molecules such as surfactants. The aim of this study was to investigate the influence of TNW mass concentration, the effect of different surfactant molecular structures (number of positively charged head groups and hydrophobic chains) as well as the aggregation state (monomers and micelles) on the stability of TNWs in two media, water and aqueous electrolyte solution of sodium bromide, thus increasing the complexity of the investigated systems.

The increase in TNW mass concentration was found to lead to less stable TNW dispersions. The effect is more pronounced when NaBr is introduced in comparison to systems in water. The surfactant molecules (DTAB and 12-2-12) alter TNW stability, thus enabling stability/aggregation under specific conditions by a delicate interplay between electrostatic repulsion, hydrophobic interactions and the structure of the surfactants. 12-2-12 proved to be much more efficient in stabilizing TNW dispersions compared to DTAB. In addition, as the mass concentration of the TNWs was increased, the TNW/surfactant systems tended to aggregate. The addition of NaBr plays a significant role in enhancing the aggregation in TNW/surfactant systems at lower concentration for both surfactants. At higher surfactant concentrations, the interaction between the TNW surface and surfactant molecules prevails. The proposed SCM accounted for the difference in molecular structure of the surfactant–TNW surface reactions. The proposed interfacial equilibrium was found to successfully describe all the experimental results. To the best of our knowledge, for the first time, a combined experimental and theoretical approach was used for such systems. Within the SCM framework, the experimental results were confirmed and 12-2-12 was found to be the surfactant with a higher affinity for TNW surfaces as compared to DTAB. The experimental data and model together with the surfactant concentration and pH can be used as a tool for tailoring the stability of NM dispersions, which is of special importance for understanding their fate in aqueous environments. The experiments combined with the modelling approach yield insight into interactions in systems that are often found in the aquatic environment, thus enabling the prediction and the optimization of TNW interaction with surfactants for their successful application but also for their removal.

## Experimental

### Materials

Dodecyltrimethylammonium bromide (DTAB, Sigma-Aldrich, 99%) was commercially obtained and recrystallized from acetone. Bis(*N,N*-dimethyl-*N*-dodecyl)ethylene-1,2-diammonium dibromide (12-2-12) was synthesized, purified and characterized as described elsewhere [33]. The TNWs were prepared using an alkaline hydrothermal procedure similar to Kasuga et al [65]. After synthesis, the TNWs were washed, filtered and dried for further analysis. The details can be found elsewhere [48]. Hydrobromic acid was provided by Kemika. Five standard buffers of pH 2, 4, 6, 8 and 10 were purchased from Riedel-de Haën and used for pH-electrode calibration. All other chemicals used in this investigation were purchased from Fluka and dissolved in Milli-Q water.

### Sample preparation

#### Preparation of neat surfactant solutions

Stock solutions of DTAB, *c*(DTAB) = 1 × 10^−1^ mol dm^−3^ and 12-2-12, *c*(12-2-12) = 1 × 10^−2^ mol dm^−3^, were prepared by dissolving dried chemicals in Milli-Q water.

#### Preparation of TNW dispersions

A stock TNW dispersion was prepared by suspending dry TNW powder in degassed Milli-Q water. The mass concentration of the stock TNW dispersion was γ = 1 g dm^−3^. The stock dispersion was sonicated using a bath sonicator (Grant, Xuba1) for 30 minutes to disperse large agglomerates and to obtain a homogenous dispersion. Dispersions containing different TNW concentrations, as shown in Table 1, were prepared by dilution of the TNW stock dispersion in Milli-Q water or in 1 × 10^−3^ mol dm^−3^ NaBr to enable the comparison of experimental and modelling results.

#### Preparation of TNW dispersions with surfactants

In order to be able to assess the influence of different surfactant aggregation states (monomers and micelles) on the TNW stability, surfactant concentrations below (monomers) and above the critical micelle concentration (micelles) were selected based on surface tension measurements (Figure S1, Table S1, Supporting Information File 1). It should be noted that in micellar surfactant solutions, monomers and micelles coexist in dynamic equilibrium [26]. In Table 3 the compositions of TNW/surfactant systems used in this study are given. The TNW/surfactant systems were prepared in Milli-Q water and in 1 × 10^−3^ mol dm^−3^ NaBr solution. In the text the systems with NaBr are labeled with asterisk, e.g., A1*.

**Table 3:** Composition of the investigated TNWs and TNW/surfactant systems and used notation. Surfactant aggregation states are also indicated. The TNW/surfactant systems were prepared in Milli-Q water or in 1 × 10^−3^ mol dm^−3^ NaBr. In the text, the systems with NaBr are labeled with asterisk, e.g., A1*.

	Milli-Q water/NaBr								
	10^4 ^*c*(DTAB) / mol dm^−3^					10^5 ^*c*(12-2-12) / mol dm^−3^			
γ(TNW) / g dm^−3^	0	1 monomers	5 monomers	50 monomers	500 micelles	1 monomers	5 monomers	50 monomers	500 micelles

0.01	CS1	A1	A2	A3	A4	D1	D2	D3	D4
0.05	CS2	B1	B2	B3	B4	E1	E2	E3	E4
0.1	CS3	C1	C2	C3	C4	F1	F2	F3	F4

### Methods

#### Characterization of surfactants

The cmc of DTAB and 12-2-12 was determined by surface tension (σ) measurements using the Du Noüy ring method (Interfacial Tensiometer K100, Krüss, Germany). The details can be found in Figure S1, Supporting Information File 1.

#### Characterization of TNWs

Powder diffraction data were collected by the PANalytical X’Pert XCharge diffractometer in the Bragg-Brentano geometry mode using Cu Kα radiation (λ = 0.154056 nm) at room temperature. PXRD patterns were scanned in the range 2θ = 5–70° with a step size of 0.08° and 10 s per step. The PANalytical High Score Plus software suite was used for data treatment. Infrared (FTIR) spectra were recorded on a Perkin Elmer FT-IR C89391 instrument at room temperature in the wavenumber range 4000–400 cm^−1^. The resolution of the FTIR spectrophotometer was 2 cm^−1^. Raman spectra were recorded on an EQUINOX 55 device equipped with an Nd:YAG laser (λ = 1064 nm) at room temperature applying a laser power of 100 mW. The resolution of the Raman spectrometer was 4 cm^−1^. The morphology of the TNWs was visualized by using high-resolution scanning electron microscopy Zeiss HR-SEM (Gemini Class) at 3–5 kV. AFM imaging was performed with a Nanosurf Flex AFM in dynamic force mode (simultaneously acquiring topography, amplitude and phase images) under ambient conditions. The TNW specific surface area (*s*) was determined via the multipoint Brunauer–Emmett–Teller method (BET) using N_2_ at 77 K and relative pressure in the range 0.05–0.3 (Micrometrics Instrument Corporation, Gemini V series surface area analyzer). A more detailed description of the experimental setup is given in Supporting Information File 1.

#### Dynamic and electrophoretic light scattering measurements

The particle sizes (*d*_h_) and zeta potentials (ζ) in TNW dispersions were determined by DLS and ELS, respectively, using a Zetasizer Nano ZS device (Malvern Instruments, Malvern, UK) equipped with a 532 nm green-light-emitting laser. The intensity of scattered light was detected at a backscattering angle of 173° to reduce multiple scattering as well as the effects of dust. To avoid overestimation arising from the scattering of larger particles, *d*_h_ was obtained as the value at peak maximum of the volume size distribution. The reported results correspond to the average of six measurements. The zeta potential of the particles was calculated from the measured electrophoretic mobility by means of the Henry equation using the Smoluchowski approximation (*f*(κa) = 1.5). The results are reported as an average value of three measurements. The data processing was done by the Zetasizer software 6.32 (Malvern Instruments). Prior to the measurements the vials were gently shaken to generate a homogenous dispersion. Both DLS and ELS measurements were performed at predetermined times of *t* = 0, 1, 4 and 24 hours at θ = 25 °C.

#### pH effect

The pH effect on the zeta potential of the TNWs in the absence (determination of the pH_iep_) and presence of the surfactants was tested as follows. The initial pH (pH_init_ ≈3) of the TNW dispersions (γ = 1 × 10^−2^ g dm^−3^) with and without the addition of surfactant (DTAB or 12-2-12) was adjusted with 1 × 10^−1^ mol dm^−3^ HBr. The dispersions were sonicated for 15 minutes using a bath sonicator (Grant, Xuba1) before zeta potential measurements. The TNW dispersions were titrated with NaOH (*c* = 1 × 10^−1^ mol dm^−3^) and left to equilibrate for 5 minutes after each addition of titrant in order to obtain stable pH electrode response. The pH was recorded using a pH meter (827 pH Lab, Metrohm) equipped with a combined glass electrode (6.0228.010, Metrohm) which was calibrated with five standard buffers (pH 2, 4, 6, 8 and 10). During the experiments, magnetic stirring was applied in order to prevent sedimentation. The experiments were carried out under inert N_2_ atmosphere and at θ = 25 °C.

#### Surface complexation modelling

The charging behavior and surface protonation constants for the TNWs in the absence and presence of surfactants have been previously determined by the surface complexation model (SCM) [58]. Details for the determination of the surface protonation constants of the TNWs can be found in our previous work [48]. Based on this, for the systems containing surfactants, a model was designed involving a basic Stern layer model with a generic surface site which interacts with positively charged surfactants, DTAB and 12-2-12. For DTAB a simple outer-sphere mechanism was applied to describe the measurement data. A similar model was applied in the case of the 12-2-12 surfactant. Two sites were introduced in the outer-sphere complex between the TNW surface and 12-2-12 due to the two positively charged polar heads of 12-2-12.

## Supporting Information

Characterization of surfactants; Characterization of TNWs; SEM micrographs of TNWs in dispersions with DTAB/12-2-12; Colloidal stability of TNWs and TNW/surfactant dispersions.

File 1 Additional experimental details.
