# Supplementary material for: Tailoring the stability/aggregation of one-dimensional TiO2(B)/titanate nanowires using surfactants
Source: Beilstein J Nanotechnol. 2019 May 13;10:1024–37. doi: 10.3762/bjnano.10.103 (PMC6541377; doi:10.3762/bjnano.10.103)
Supplement: File 1 — Additional experimental details. [file Beilstein_J_Nanotechnol-10-1024-s001.pdf]

BEILSTEIN JOURNAL OF NANOTECHNOLOGY

# Supporting Information

for

# Tailoring the stability/aggregation of one-dimensional $TiO_2$(B)/titanate nanowires using surfactants

Atiđa Selmani, Johannes Lützenkirchen, Kristina Kučanda, Dario Dabić, Engelbert Redel, Ida Delač Marion, Damir Kralj, Darija Domazet Jurašin and Maja Dutour Sikirić

*Beilstein J. Nanotechnol.* **2019,** *10,* 1024–1037. doi:10.3762/bjnano.10.103

# Additional experimental details

License and Terms: This is a supporting information file under the terms of the Creative Commons Attribution License (http://creativecommons.org/licenses/by/4.0). Please note that the reuse, redistribution and reproduction in particular requires that the authors and source are credited. The license is subject to the *Beilstein Journal of Nanotechnology* terms and conditions: (https://www.beilstein-journals.org/bjnano)

## 1. Molecular structures of monomeric and dimeric surfactants used in this study

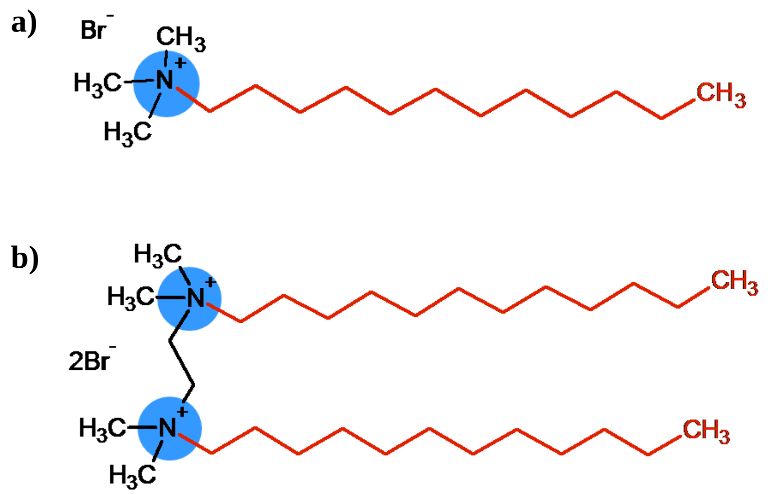


Scheme S1: Molecular structures of a) monomeric, dodecyltrimethylammonium bromide (DTAB) and its corresponding b) dimeric, bis(*N*,*N*-dimethyl-*N*-dodecyl)ethylene-1,2-diammonium dibromide (12-2-12) surfactant.

## 2. Characterization of surfactants

### 2.1. Surface tension measurements

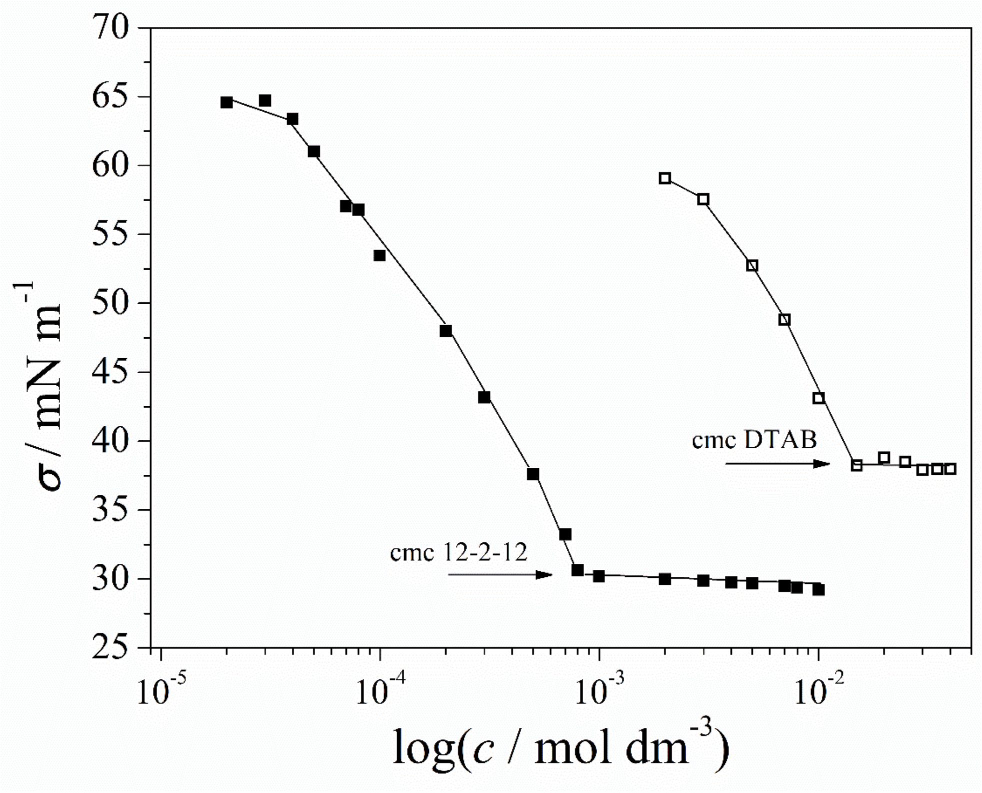


Figure S1: Variation of the surface tension ($\sigma$) with the surfactant concentration ($c$) for DTAB (□) and gemini, 12-2-12 (■) surfactants at $\vartheta$ = (25 ± 0.1) °C.

Table S1: The critical micelle concentration (cmc) determined by surface tension measurements (cmc) for DTAB and gemini, 12-2-12 surfactants at $\vartheta$ = (25 ± 0.1) °C.

| Surfactant | $10^3$ cmc(Milli-Q water) / mol dm$^{-3}$ | $10^3$ cmc(NaBr) / mol dm$^{-3}$ |
|---|---|---|
| DTAB | 14.2 | 14.1 |
| 12-2-12 | 0.80 | 0.83 |

The cmcs of DTAB and 12-2-12 were determined by surface tension ($\sigma$) measurements using the Du Noüy ring method (Interfacial Tensiometer K100, Krüss,

Germany). These values were then corrected by using the tables of Huh and Mason [S1]. The surface tension of water was measured regularly in order to provide values for the pure solvent and to check that the technique delivered reliable results. All measurements were conducted at $\vartheta = (25 \pm 0.1)$ °C.

# 3. Characterization of TNWs

## 3.1. Structure and composition of TNWs

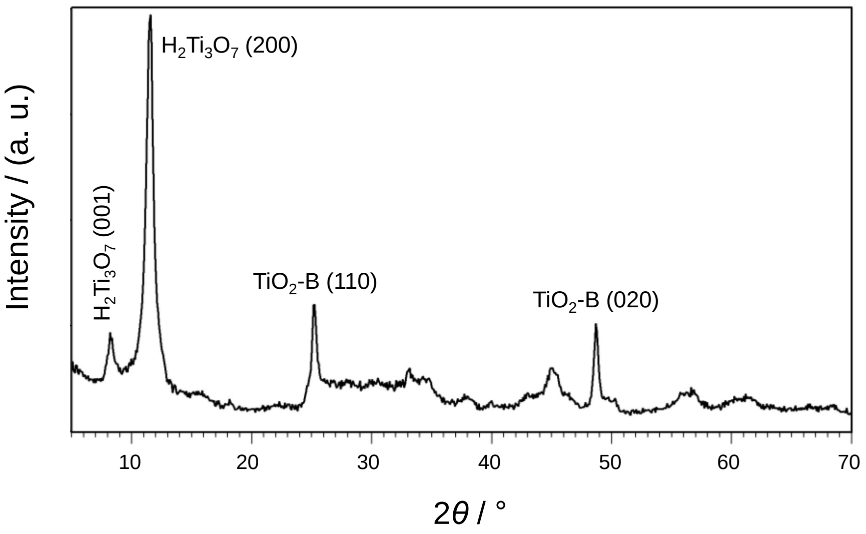


Figure S2: Powder X-ray diffraction pattern of the synthesized TNWs.

The structure of the TNWs was studied by X-ray powder diffraction (PXRD). The diffraction peaks at the $2\theta$ positions 8.3°, 11.5°, correspond to (001) and (200) reflections, characteristic for layered titanates ($H_2Ti_3O_7$) as represented on Figure S2. Similar patterns were obtained by Selmani *et al.* [S3], Bavykin and Walsh [S4] and Kolen'ko *et al.* [S5]. Peaks observed at the $2\theta$ positions 24.9° (110) and 48.6° (020) were attributed to $TiO_2$-B phase (JCPDS 74−1940) [S6,S7].

PXRD results were confirmed by FT-IR and Raman spectroscopy (Figure S3). The FT-IR spectrum revealed absorption bands at 3131, 2371, 1627, 925, 733 and 460 cm$^{-1}$ while absorption bands at 671, 599, 519, 438, 344, 267, 189 and 149 cm$^{-1}$ were observed in the

Raman spectrum. The obtained data are in good agreement with previously published results for trititanate layered TNWs [S3,S8].

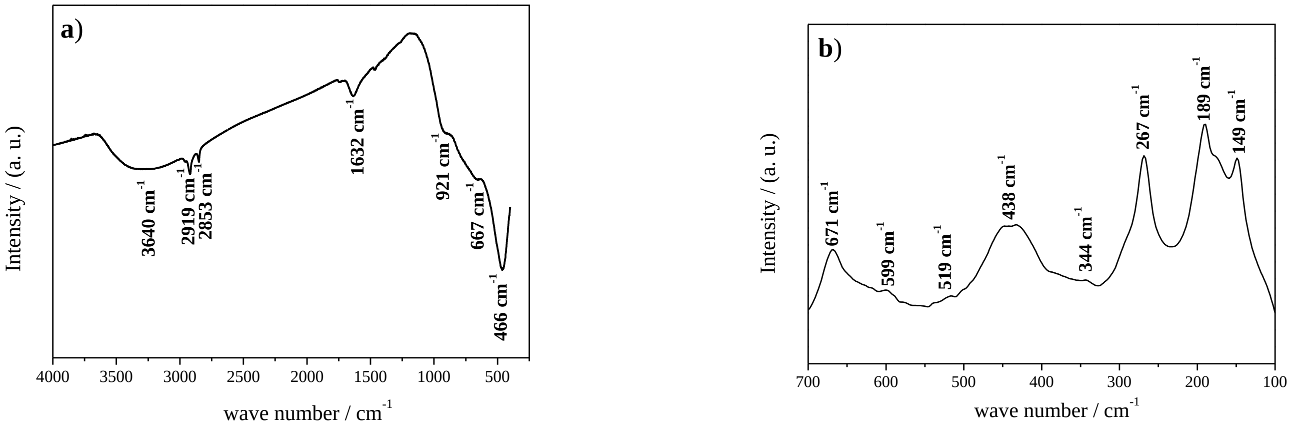


Figure S3: Vibrational spectra of synthesized TNWs: **a)** FT-IR and **b)** Raman.

## 3.2. Atomic force microscopy

Atomic force microscopy (AFM) imaging was performed with a Nanosurf Flex AFM in dynamic force mode (simultaneously acquiring topography, amplitude and phase images) under ambient conditions. AFM probes were obtained from AppNano (ACLA silicon tips of nominal spring constant 20–95 N $m^{-1}$ with tip radius less than 10 nm and nominal resonant frequency of 145–230 kHz). During the measurement, the vibration frequency was 189 kHz with a vibration amplitude of 150 mV. Subsequently, AFM images were processed by the Gwyddion software [S9].

## 3.3. Scanning electron microscopy (SEM)

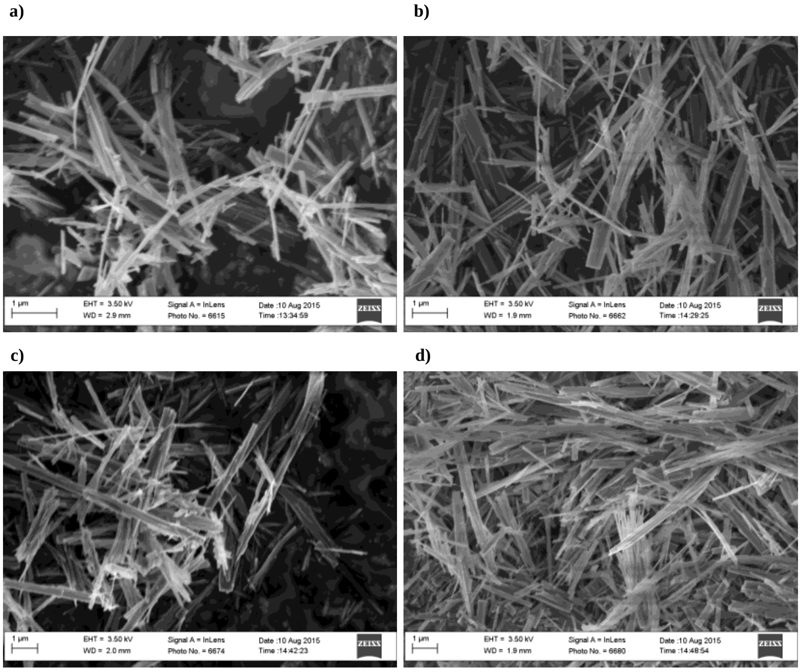


Figure S4: Micrographs of TNW in dispersions with DTAB taken by scanning electron microscopy: **a)** $c$(DTAB) / mol dm$^{-3}$ = 1 × 10$^{-4}$, **b)** $c$(DTAB) / mol dm$^{-3}$ = 5 × 10$^{-4}$, **c)** $c$(DTAB) / mol dm$^{-3}$ = 5 × 10$^{-3}$, **d)** $c$(DTAB) / mol dm$^{-3}$ = 5 × 10$^{-2}$.

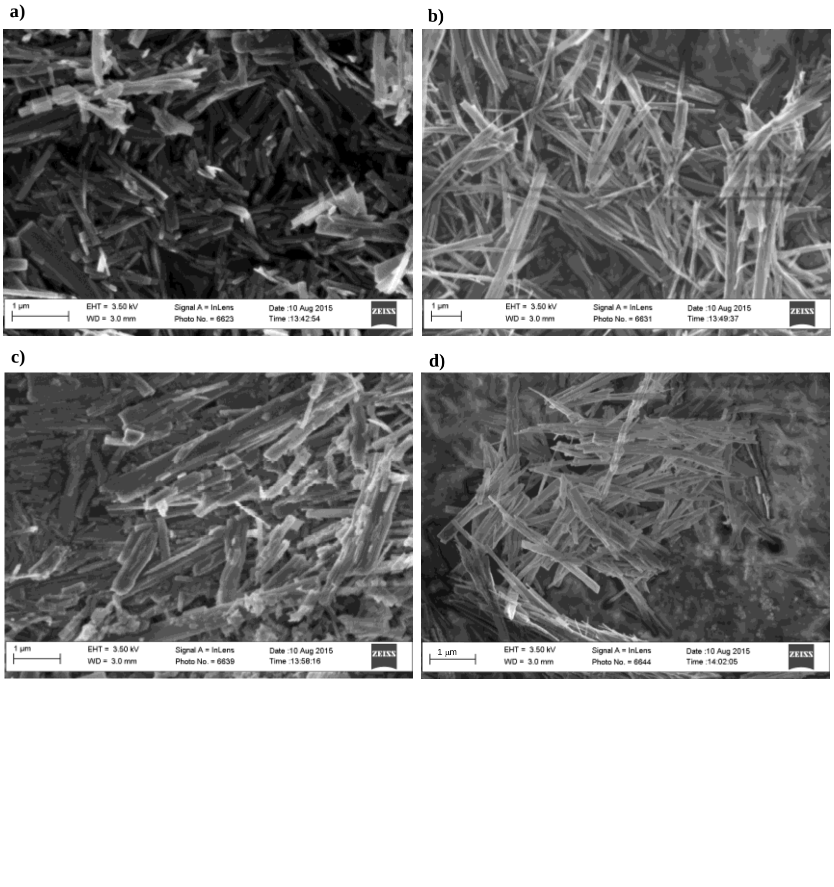

Figure S5: Micrographs of TNW in dispersions with 12-2-12 taken by scanning electron microscopy: **a)** *c*(12-2-12)/mol dm$^{-3}$ = 1 × 10$^{-5}$, **b)** *c*(12-2-12)/mol dm$^{-3}$ = 5 × 10$^{-5}$, **c)** *c*(12-2-12)/mol dm$^{-3}$ = 5 × 10$^{-4}$, **d)** *c*(12-2-12)/mol dm$^{-3}$ = 5 × 10$^{-3}$.

## 4. Stability of TNW and TNW/surfactant dispersions

The stability of the TNW dispersions was monitored by visual observation. Visual observation of the TNW dispersions is displayed in Figure S6. Immediately after mixing all 3 TNW dispersions, without added surfactant, were stable and no phase separation was observed based on visual inspection (Figure S6). However, after 24 h small amounts of white sediment were present in all control systems. Phase separation was most pronounced in the system with the lowest TNW concentration (CS1). The aggregation behaviour of TNWs in the presence of monomer (1-3) and micellar (4) DTAB concentrations was monitored over 24 h. The results of the visual monitoring are presented in Figure S6. Immediately after preparation ($t$ = 0 h) all systems retain their stability as no phase separation was observed by visual inspection. Conversely, after 24 h aging time significant differences in the stability of dispersions were detected. The turbidity decrease due to the formation of white sediments was most remarkable in the systems with the lowest TNW mass concentration (A1-A4). Furthermore, it seems that only in the presence of the lowest monomer DTAB concentration the TNWs are stabilized as compared to the corresponding control systems. Higher monomer DTAB concentrations promoted aggregation in all investigated systems. The most pronounced phase separation was visible in systems with the highest monomer DTAB concentration (B3, C3). However, in the presence of DTAB micellar concentration (B4, C4) TNWs aggregated to a lesser extent than in B3 and C3.

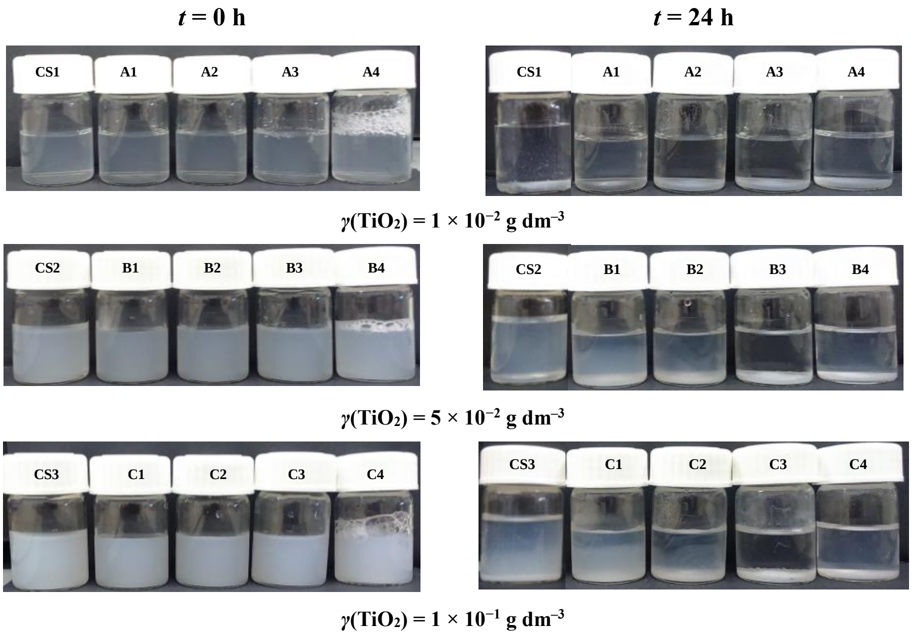


Figure S6: Visual observations of the TNW/DTAB systems in Milli-Q water immediately after mixing and after 24 h aging time; *c*(DTAB)/mol dm$^{-3}$ = 0 (**CS**), 1 × 10$^{-4}$ (**1**), 5 × 10$^{-4}$ (**2**), 5 × 10$^{-3}$ (**3**), 5 × 10$^{-2}$ (**4**).

In order to elucidate the effect of different molecular structure, the influence of the gemini analogue of DTAB, 12-2-12 surfactants on TNWs stability was investigated. In Figure S7 results of visual inspection of TNW dispersions with and without dimeric surfactant immediately after mixing (*t* = 0 h) and after aging one day are shown. The white colloidal dispersions of all TNW/12-2-12 systems were stable immediately after mixing, *i.e*. no phase separation was visible. However, after 24 h aging, depending on the 12-2-12 concentration, significant differences were observed in dispersions stability. In systems with lowest TNW concentration, phase separation was pronounced in D1 and D2 while in D3 and D4 phase separation occurred to a lesser extent. For 5-fold higher TNW concentrations (E1-E4) after 24 h TNWs retained their stability only in systems with lowest and highest 12-2-12 concentrations (E1, E3 and E4). Inspection of E1, E3 and E4 revealed only partial phase separation. E2 was destabilized after 24 h and resulted in the dispersion of lowest turbidity.

Visually very similar trends in colloidal stability of TNWs regarding 12-2-12 concentrations were also observed in systems containing the highest TNW concentration (F1–F4). It should be noted that most pronounced aggregation and phase separation in TNW/DTAB systems occurred at 100-fold higher surfactant concentration in comparison to 12-2-12.

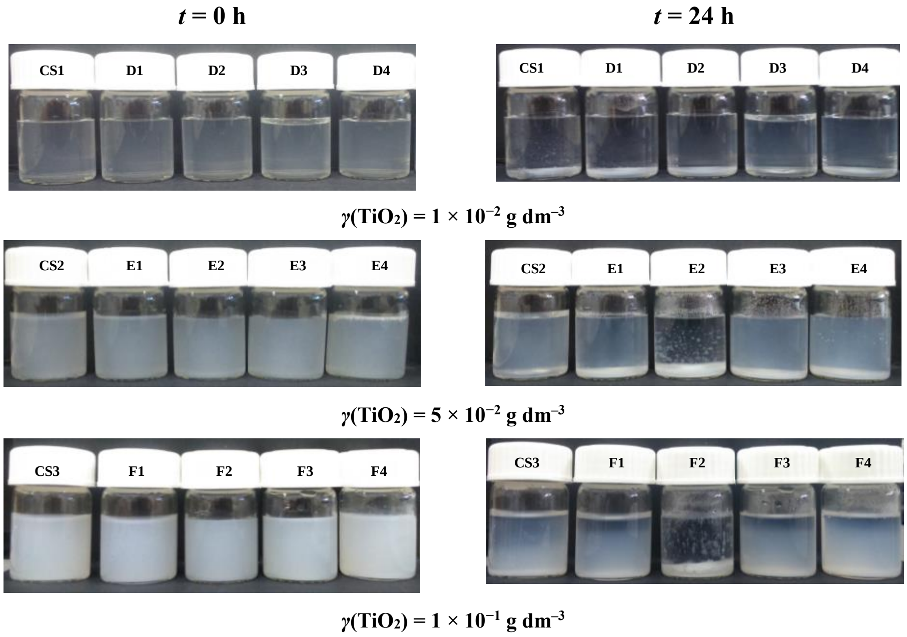


Figure S7: Visual observations of the TNW/12-2-12 systems in Milli-Q water immediately after mixing and after 24 h aging time; $c$(12-2-12)/mol dm$^{-3}$ = 0 (**CS**), 1 × 10$^{-5}$ (**1**), 5 × 10$^{-5}$ (**2**), 5 × 10$^{-4}$ (**3**), 5 × 10$^{-3}$ (**4**).

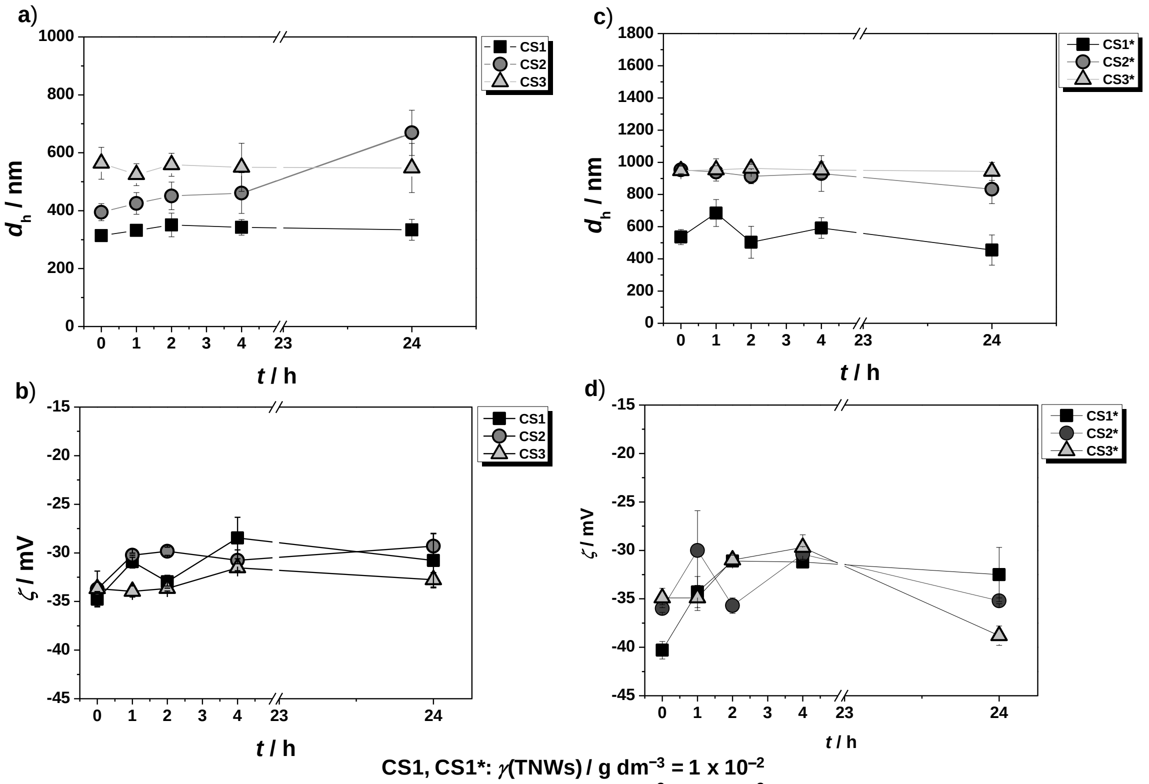


**CS1, CS1*: $\gamma$(TNWs) / g dm$^{-3}$ = 1 x 10$^{-2}$**
**CS2, CS2*: $\gamma$(TNWs) / g dm$^{-3}$ = 5 x 10$^{-2}$**
**CS3, CS3*: $\gamma$(TNWs) / g dm$^{-3}$ = 1 x 10$^{-1}$**

Figure S8: Variation of hydrodynamic diameter ($d_h$) and zeta potential ($\zeta$) of uncoated TNWs during time in **a)**, **b)**, Milli-Q water and **c)**, **d),** $1 \times 10^{-3}$ mol dm$^{-3}$ NaBr. $\gamma$(TNWs)/g dm$^{-3}$ = $1 \times 10^{-2}$ (**CS1**), $5 \times 10^{-2}$ (**CS2**), $1 \times 10^{-1}$ (**CS3**). $\vartheta$ = 25 °C. The systems with NaBr are labeled with asterisk, *.

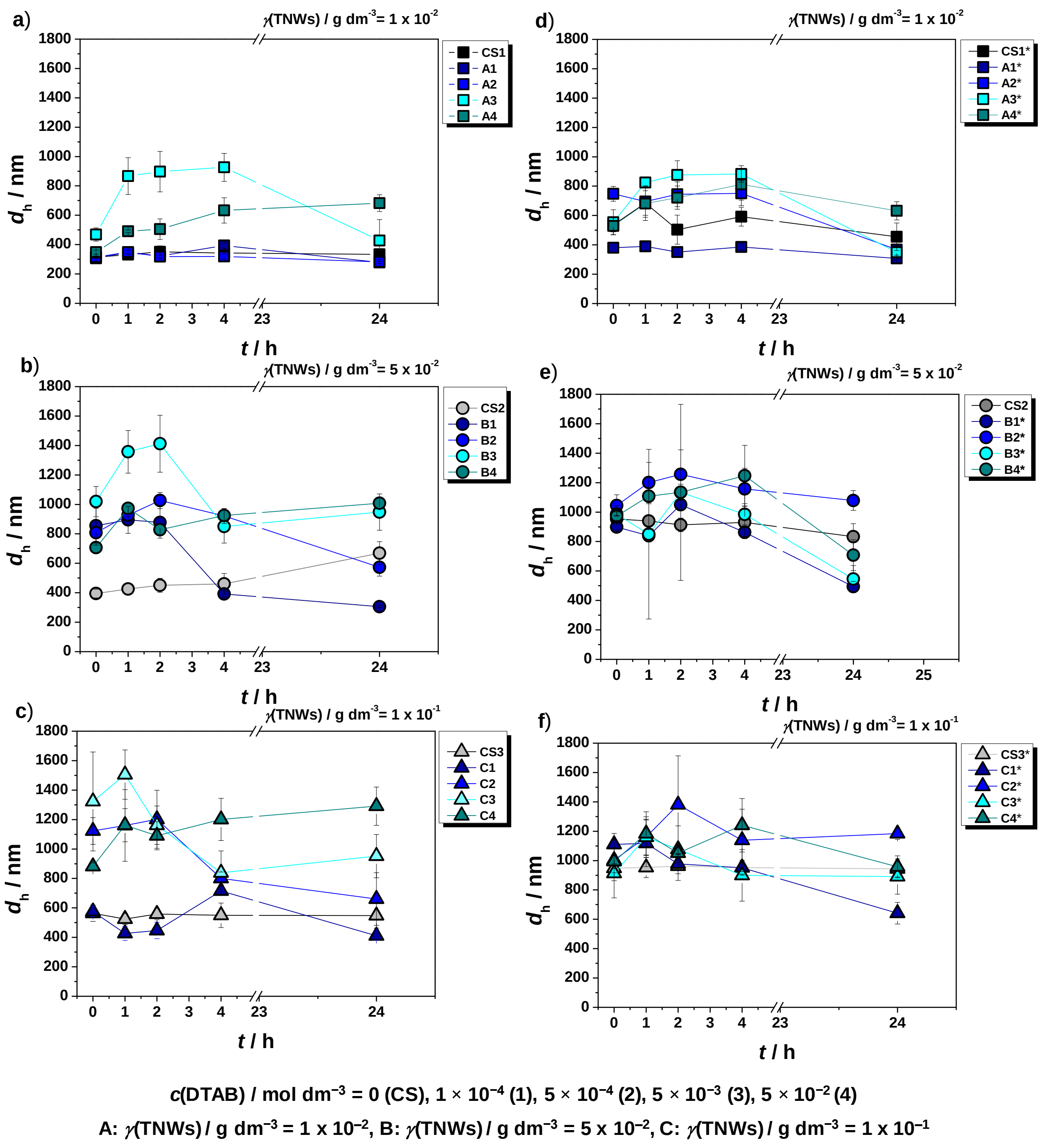


Figure S9: Variation of hydrodynamic diameter ($d_h$) in TNW/DTAB systems during time in **a)**, **b)**, **c)** Milli-Q water and **d)**, **e)**, **f)** NaBr solution, $c$(NaBr)/mol dm$^{-3}$ = 1 × 10$^{-3}$. $c$(DTAB) / mol dm$^{-3}$ = 0 (**CS**), 1 × 10$^{-4}$ (**1**), 5 × 10$^{-4}$ (**2**), 5 × 10$^{-3}$ (**3**), 5 × 10$^{-2}$ (**4**). $\vartheta$ = 25 °C. The systems with NaBr are labeled with asterisk, *.

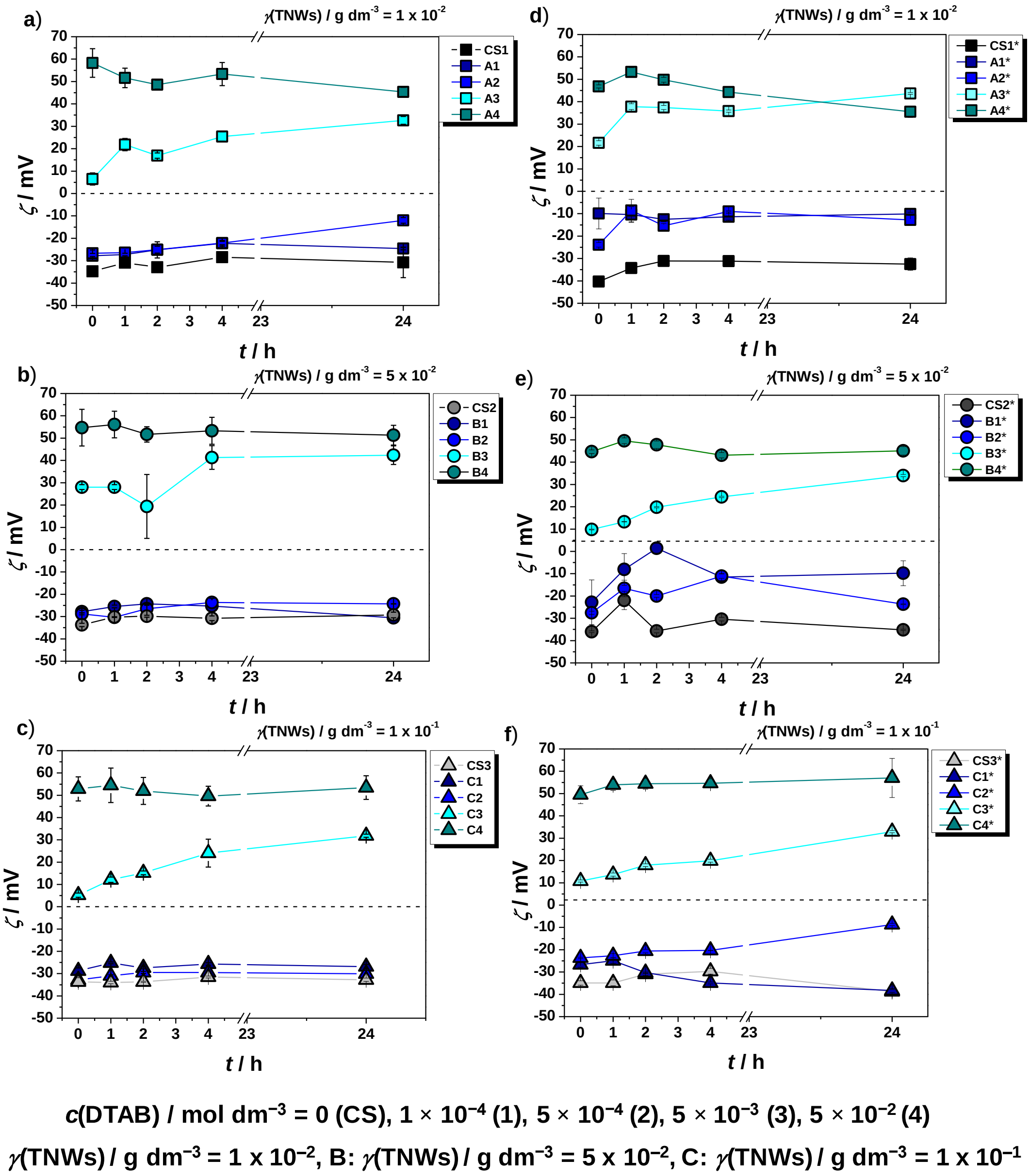


Figure S10: Variation of zeta potential ($\zeta$) in TNW/DTAB systems during time in **a)**, **b)**, **c)** Milli-Q water and **d)**, **e)**, **f)** NaBr solution, $c$(NaBr)/mol dm$^{-3}$ = 1 × 10$^{-3}$. $c$(DTAB) / mol dm$^{-3}$ = 0 (**CS**), 1 × 10$^{-4}$ (**1**), 5 × 10$^{-4}$ (**2**), 5 × 10$^{-3}$ (**3**), 5 × 10$^{-2}$ (**4**). $\vartheta$ = 25 °C. The systems with NaBr are labeled with asterisk, *.

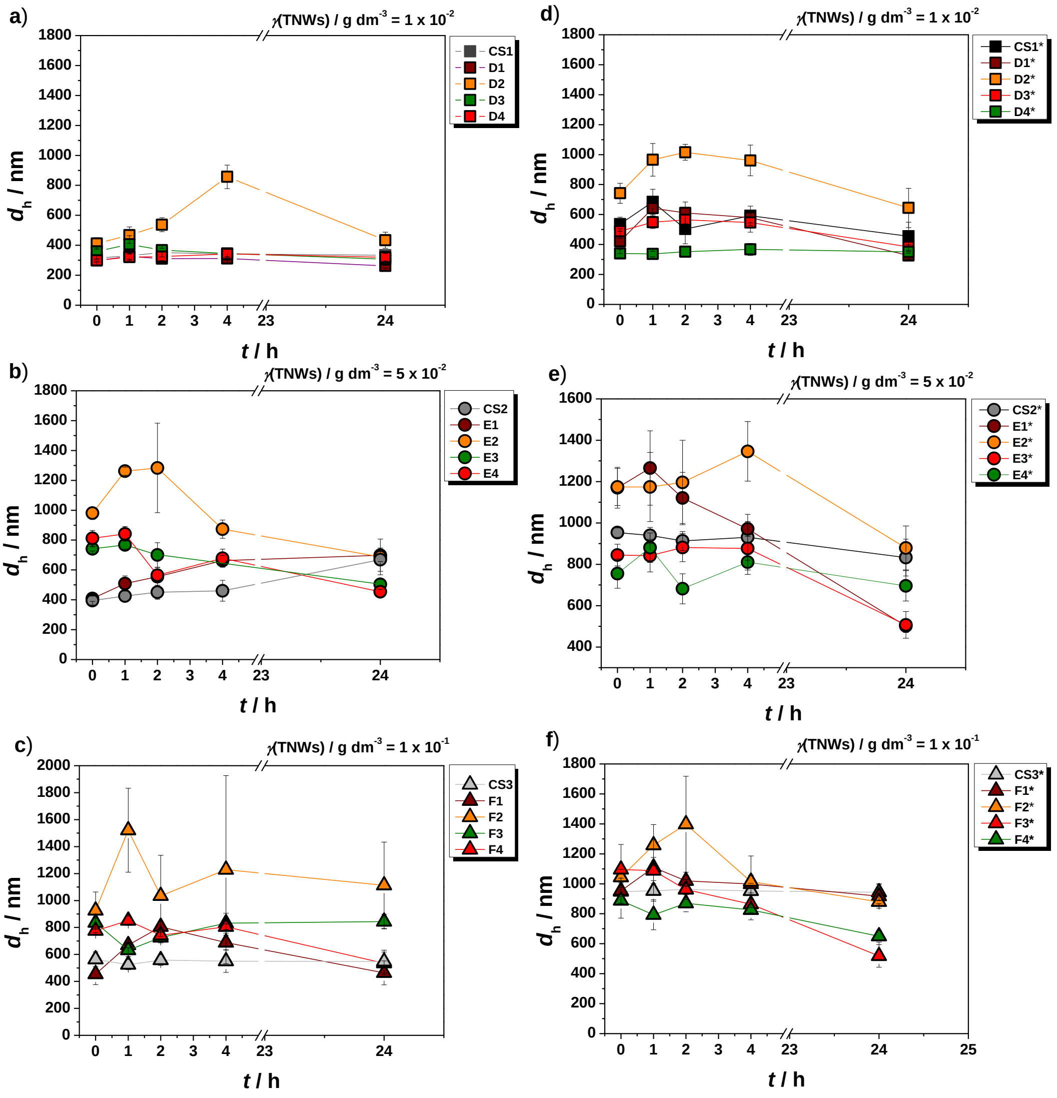


**$c$(12-2-12) / mol dm$^{-3}$ = 0 (CS), 1 × 10$^{-5}$ (1), 5 × 10$^{-5}$ (2), 5 × 10$^{-4}$ (3), 5 × 10$^{-3}$ (4)**

**A: $\gamma$(TNWs) / g dm$^{-3}$ = 1 x 10$^{-2}$, B: $\gamma$(TNWs) / g dm$^{-3}$ = 5 x 10$^{-2}$, C: $\gamma$(TNWs) / g dm$^{-3}$ = 1 x 10$^{-1}$**

Figure S11: Variation of hydrodynamic diameter ($d_h$) in TNW/12-2-12 systems during time in **a)**, **b)**, **c)** Milli-Q water and **d)**, **e)**, **f)** NaBr solution, $c$(NaBr)/mol dm$^{-3}$ = 1 × 10$^{-3}$. $c$(12-2-12) / mol dm$^{-3}$ = 0 (**CS**), 1 × 10$^{-5}$ (**1**), 5 × 10$^{-5}$ (**2**), 5 × 10$^{-4}$ (**3**), 5 × 10$^{-3}$ (**4**). $\vartheta$ = 25 °C. The systems with NaBr are labeled with asterisk, *.

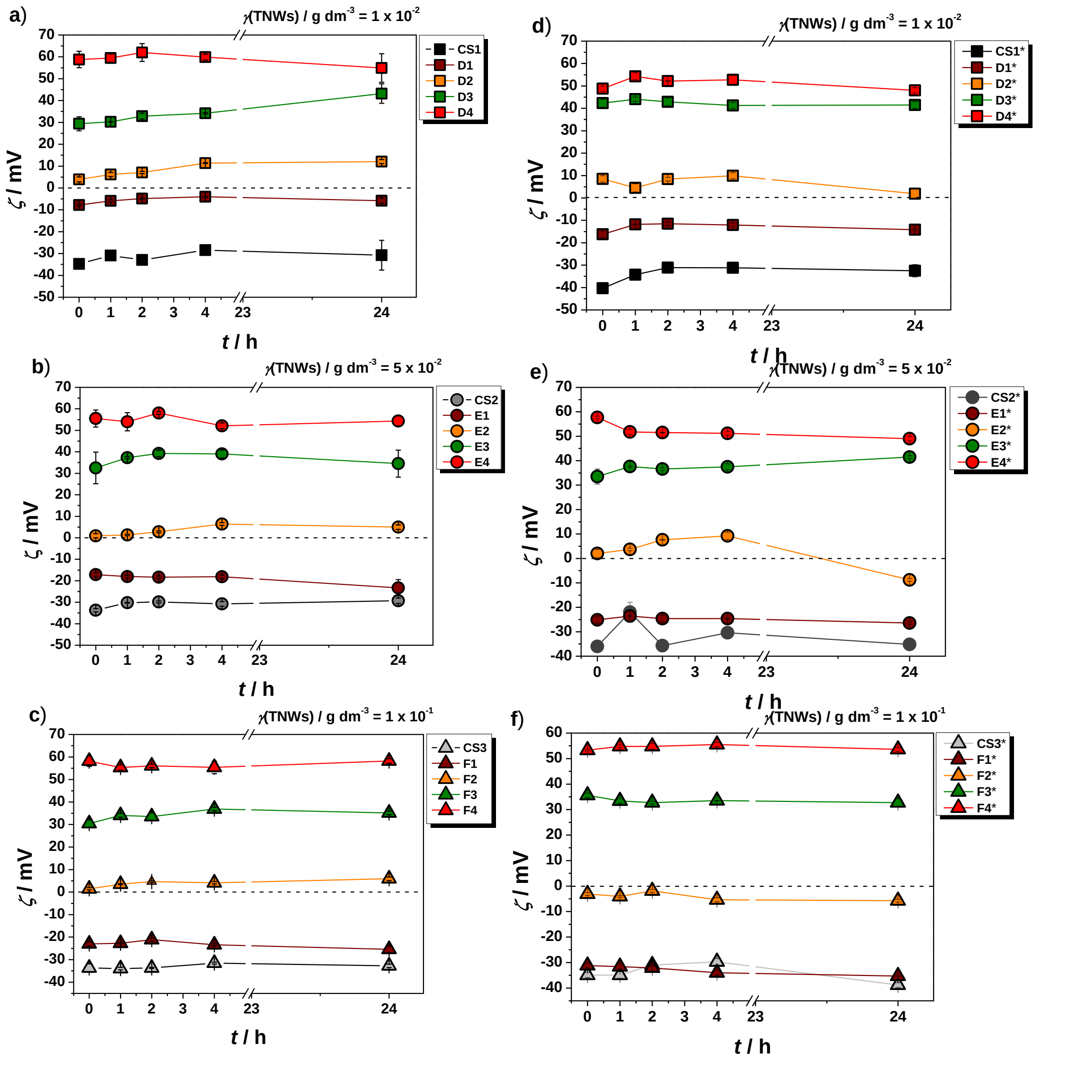


**$c$(12-2-12) / mol dm$^{-3}$ = 0 (CS), 1 × 10$^{-5}$ (1), 5 × 10$^{-5}$ (2), 5 × 10$^{-4}$ (3), 5 × 10$^{-3}$ (4)**

**A: $\gamma$(TNWs) / g dm$^{-3}$ = 1 x 10$^{-2}$, B: $\gamma$(TNWs) / g dm$^{-3}$ = 5 x 10$^{-2}$, C: $\gamma$(TNWs) / g dm$^{-3}$ = 1 x 10$^{-1}$**

Figure S12: Variation of zeta potential ($\zeta$) in TNW/12-2-12 systems during time in **a)**, **b)**, **c)** Milli-Q water and **d)**, **e)**, **f)** NaBr solution, $c$(NaBr)/mol dm$^{-3}$ = 1 × 10$^{-3}$. $c$(12-2-12) / mol dm$^{-3}$ = 0 (**CS**), 1 × 10$^{-5}$ (**1**), 5 × 10$^{-5}$ (**2**), 5 × 10$^{-4}$ (**3**), 5 × 10$^{-3}$ (**4**). $\vartheta$ = 25 °C. The systems with NaBr are labeled with asterisk, *.

## 5. References


[S1] C. Huh and S. G. Mason, *Colloid Polym. Sci.*, 1975, **253**, 566–580.

[S2] M. Rodahl, F. Höök, A. Krozer, P. Brzezinski and B. Kasemo, *Rev. Sci. Instrum.*, 1995, **66**, 3924–3930.

[S3] A. Selmani, M. Špadina, M. Plodinec, I. Delač Marion, M. G. Willinger, J. Lützenkirchen, H. D. Gafney and E. Redel, *J. Phys. Chem. C*, 2015, **119**, 19729–19742.

[S4] D. V. Bavykin and F. C. Walsh, *J. Phys. Chem. C*, 2007, **111**, 14644–14651.

[S5] Y. V. Kolenko, K. a. Kovnir, A. I. Gavrilov, A. V. Garshev, J. Frantti, O. I. Lebedev, B. R. Churagulov, G. Van Tendeloo and M. Yoshimura, *J. Phys. Chem. B*, 2006, **110**, 4030–4038.

[S6] G. Xiang, Y. G. Wang, J. Li, J. Zhuang and X. Wang, *Sci Rep*, 2013, **3**, 1411.

[S7] J. Li, W. Wan, H. Zhou, J. Li and D. Xu, *Chem. Commun. (Camb).*, 2011, **47**, 3439–41.

[S8] T. Kasuga, M. Hiramatsu, A. Hoson, T. Sekino and K. Niihara, *Langmuir*, 1998, **14**, 3160–3163.

[S9] D. Nečas and P. Klapetek, *Open Phys.*, 2012, **10**, 181–188.
